# Systematic optimization of prime editing for the efficient functional correction of *CFTR* F508del in human airway epithelial cells

**DOI:** 10.1038/s41551-024-01233-3

**Published:** 2024-07-10

**Authors:** Alexander A. Sousa, Colin Hemez, Lei Lei, Soumba Traore, Katarina Kulhankova, Gregory A. Newby, Jordan L. Doman, Keyede Oye, Smriti Pandey, Philip H. Karp, Paul B. McCray, David R. Liu

**Affiliations:** 1 Merkin Institute of Transformative Technologies in Healthcare, The Broad Institute of MIT and Harvard, Cambridge, MA USA; 2 Department of Chemistry and Chemical Biology, Harvard University, Cambridge, MA USA; 3 Howard Hughes Medical Institute, Harvard University, Cambridge, MA USA; 4 Stead Family Department of Pediatrics and Pappajohn Biomedical Institute, Roy J. and Lucille A. Carver College of Medicine, University of Iowa, Iowa City, IA USA; 5 Department of Genetic Medicine, Johns Hopkins University School of Medicine, Baltimore, MD USA; 6 Department of Biomedical Engineering, Johns Hopkins University, Baltimore, MD USA; 7 Department of Internal Medicine and Pappajohn Biomedical Institute, Roy J. and Lucille A. Carver College of Medicine, Iowa City, IA USA; 8 Howard Hughes Medical Institute, University of Iowa, Iowa City, IA USA

**Keywords:** Targeted gene repair, CRISPR-Cas9 genome editing

## Abstract

Prime editing (PE) enables precise and versatile genome editing without requiring double-stranded DNA breaks. Here we describe the systematic optimization of PE systems to efficiently correct human cystic fibrosis (CF) transmembrane conductance regulator (*CFTR*) F508del, a three-nucleotide deletion that is the predominant cause of CF. By combining six efficiency optimizations for PE—engineered PE guide RNAs, the PEmax architecture, the transient expression of a dominant-negative mismatch repair protein, strategic silent edits, PE6 variants and proximal ‘dead’ single-guide RNAs—we increased correction efficiencies for *CFTR* F508del from less than 0.5% in HEK293T cells to 58% in immortalized bronchial epithelial cells (a 140-fold improvement) and to 25% in patient-derived airway epithelial cells. The optimizations also resulted in minimal off-target editing, in edit-to-indel ratios 3.5-fold greater than those achieved by nuclease-mediated homology-directed repair, and in the functional restoration of CFTR ion channels to over 50% of wild-type levels (similar to those achieved via combination treatment with elexacaftor, tezacaftor and ivacaftor) in primary airway cells. Our findings support the feasibility of a durable one-time treatment for CF.

## Abstract (web summary)

A systematic implementation of six efficiency optimizations for prime editing led to high levels of functional correction, in airway epithelial cells, of the predominant mutation that causes cystic fibrosis.

## Main

Prime editing (PE) enables the replacement of targeted DNA nucleotides with any specified replacement of up to hundreds of nucleotides, thereby enabling a wide variety of substitutions, insertions and deletions in the genomes of living systems^1–4^. The mechanism of PE is inherently resistant both to bystander editing (unwanted editing at the target site)^1^ and to off-target editing (unwanted editing away from the target site)^1,3,5–17^. In contrast with nuclease-mediated gene editing, prime editors do not require the creation of double-stranded DNA breaks (DSBs), minimizing undesirable outcomes such as uncontrolled insertions and deletions (indels)^2–4,18^, large deletions^19,20^, p53 activation^21–23^, retrotransposon insertion^24^ and chromosomal defects^19,25–28^. PE does not require co-delivery of donor DNA template, is active in mitotic and non-mitotic cells^1,5,29–34^, and has been successfully performed in vivo in mice^5,29,32–38^ and in non-human primates^39^.

**Fig. 1:**
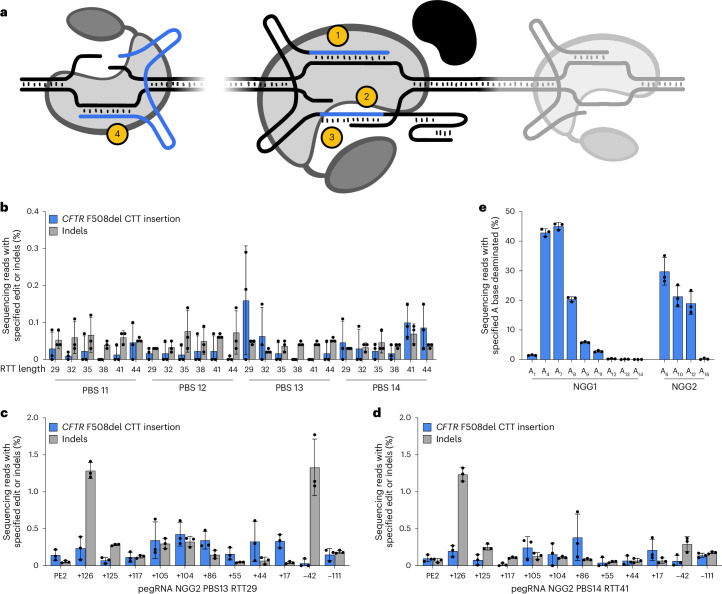
Basic PE2 and PE3 systems inefficiently correct *CFTR* F508del. **a**, Schematic of PE2 and PE3 systems. The factors that influence PE2 and PE3 editing efficiency include (1) pegRNA spacer sequence, (2) pegRNA PBS length, (3) pegRNA RTT length and (4) ngRNA spacer sequence. **b**, Quantification of PE2 correction of *CFTR* F508del in HEK293T cells using NGG2 pegRNAs with different combinations of PBS and RTT lengths, with the PBS and RTT lengths shown in nucleotides. **c**,**d**, PE3 correction of *CFTR* F508del in HEK293T cells using NGG2 PBS13 RTT29 pegRNA (**c**) or NGG2 PBS14 RTT41 pegRNA (**d**) in combination with several ngRNAs. The *x*-axis labels identify different ngRNAs by their nicking position relative to the pegRNA nick (in base pairs). The PE2 *x*-axis label specifies an editing condition with no ngRNA. **e**, Adenine base editing in HEK293T cells at adenines in the NGG1 and NGG2 protospacers. Adenines (A) are numbered 5′ to 3′ starting from the PAM-distal end of the protospacers. For **b**–**e**, the data and error bars represent the means and standard deviations, respectively, of the three independent biological replicates (shown as black dots). Source data

Prime editors combine a programmable nickase such as *Streptococcus pyogenes* Cas9 (SpCas9) H840A nickase with a reverse transcriptase (RT) such as an engineered Moloney murine leukaemia virus RT^1^. A PE guide RNA (pegRNA) guides the prime editor protein to its spacer-specified genomic target and also contains a 3′ extension with a primer binding site (PBS) complementary to the nicked target DNA and an RT template (RTT) encoding the desired edited sequence^1,40^. When bound to its programmed target sequence, the prime editor–pegRNA complex nicks the target site to create an accessible 3′ end of single-stranded DNA. This target DNA 3′ end hybridizes to the pegRNA’s 3′ PBS, creating a primer–template complex that initiates reverse transcription using the RTT to create a 3′ DNA flap containing the edited DNA sequence^1^. The 3′ flap of edited DNA can displace the original sequence and be ligated into the genome, creating a DNA heteroduplex of edited and unedited DNA strands. This editing intermediate is then resolved into a permanent edit on both strands by DNA repair or replication^41^. A nickase, RT and pegRNA constitute a ‘PE2-type’ editing system. A ‘PE3-type’ system adds an additional nicking guide RNA (ngRNA) that nicks the unedited strand of the DNA heteroduplex intermediate to enhance editing efficiency by directing mismatch repair (MMR) to remake the unedited strand using the edited strand as a template^1^ (Fig. 1a).

PE is well suited to correct pathogenic mutations such as the three-base-pair CTT deletion (F508del) in the cystic fibrosis transmembrane conductance regulator (*CFTR*) gene that results in the loss of phenylalanine 508 in the CFTR protein. This deletion is the most common cause of cystic fibrosis (CF)^42^, an autosomal recessive disorder that affects more than 160,000 people worldwide^43^. In people with CF, *CFTR* mutations impair the anion channel activity of CFTR that conducts Cl^−^ and HCO_3_^−^ transport across the apical membranes of epithelia-lined secretory organs including the pancreas, gastrointestinal tract and respiratory tract^44–48^. While over 2,000 *CFTR* variants have been identified and more than 700 are verified to cause CF, one mutation, *CFTR* F508del, is present in 85% of patients with CF^42,49^. The CFTR F508del protein misfolds, and the majority of the protein undergoes proteasomal degradation^47,50–52^. If trafficked to the cell membrane, the CFTR F508del channel is functional, albeit with a reduced open probability (*P*_o_)^53^. These molecular defects have been the target of several breakthrough small-molecule therapies that have greatly enhanced clinical outcomes for patients with CF^54–58^. While effective and impactful, current small-molecule therapies require daily administration for life at an annual cost of approximately US$300,000 (refs. ^43,59,60^). The development of a gene editing strategy to precisely correct the *CFTR* F508del CTT deletion could offer a path to a durable, one-time treatment for the most common CF-causing mutation.

Here, we describe the development of a PE approach to efficiently correct the *CFTR* F508del mutation in primary airway epithelial cells from patients with CF and rescue CFTR-dependent anion channel activity. Like others^8^, our initial attempts to correct this mutation with the originally reported PE2 and PE3 systems yielded minimal editing, revealing that this mutation was an unusually challenging one to correct by PE. By systematically applying six recent advances in PE technology, we achieved F508del correction efficiencies of up to 58% in an immortalized human bronchial epithelial cell model and 25% in primary airway epithelial cells from patients with CF. Optimized PE restored CFTR channel function to greater than 50% of wild-type levels in primary airway epithelial cells from patients with CF. These results demonstrate proof-of-concept for the direct therapeutic correction of the most common CF-causing *CFTR* mutation and serve as a roadmap for engineering PE strategies at difficult-to-edit therapeutic targets.

## Results

### Initial attempts to correct *CFTR* F508del with PE2 and PE3

**Extended Data Fig. 1:**
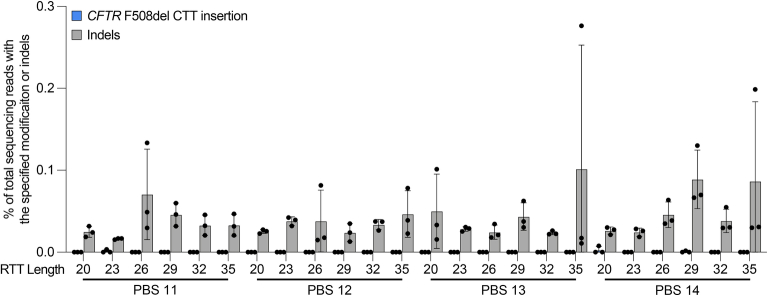
PE2 correction of *CFTR* F508del is undetectable with NGG1 pegRNAs. A panel of 24 pegRNAs with variable PBS and RTT lengths was transfected into *CFTR* F508del HEK293T cells. PBS and RTT lengths are listed in nucleotides. Data and error bars represent mean and standard deviation, respectively, collected from three independent biological replicates (shown as black dots). Source data

We sought to correct the *CFTR* F508del mutation soon after we originally reported PE in 2019 (ref. ^1^). To efficiently test and optimize PE *CFTR* F508del correction strategies^61^, we first used PE to generate a clonal HEK293T cell line homozygous for this deletion in the endogenous *CFTR* gene (Supplementary Fig. 1 and Supplementary Tables 1 and 2). Next, we used this cell line with PE2 to screen pegRNAs with combinations of PBS and RTT lengths at two F508del CTT deletion-proximal protospacers with NGG protospacer adjacent motifs (PAMs; NGG1 and NGG2; Supplementary Fig. 2). We observed no detectable correction of *CFTR* F508del using NGG1 pegRNAs (Extended Data Fig. 1), which we hypothesized was due to the presence of a TTTT sequence on the non-PAM containing strand of the NGG1 protospacer. We speculate that this TTTT may act as an RNA polymerase III transcriptional terminator that prevents complete pegRNA PBS transcription from a U6 promoter, resulting in an incompletely transcribed pegRNA that cannot support PE (Supplementary Fig. 2). We did observe F508del correction when NGG2 pegRNAs were used, but no NGG2 PE2 editing strategies yielded average editing efficiencies greater than 0.2% (Fig. 1b).

To enhance correction of F508del, we performed a PE3 screen of two NGG2 pegRNAs (NGG2 PBS13 RTT29 and NGG2 PBS14 RTT41) against a panel of all available ngRNA protospacers with pegRNA–ngRNA inter-nick distances of approximately 125 bp (Fig. 1c,d). While the best-performing PE3 NGG2 pegRNA offered up to a 2.6-fold improvement in edit installation efficiency over PE2, the maximum mean editing for any NGG2 PE3 edit did not exceed 0.5%, suggesting that PE3 systems alone cannot efficiently correct *CFTR* F508del.

We hypothesized that the inefficient correction of F508del using NGG1 and NGG2 could be due to inaccessibility of the edit site to Cas effectors due to chromatin state, which has been reported to negatively affect all major forms of mammalian cell genome editing^9,62–64^. *CFTR* resides within a topologically associated domain whose chromatin state is tightly controlled by protein interactions with numerous enhancers and insulators embedded within the gene^65^. To distinguish chromatin accessibility from prime editor-specific editing limitations, we targeted both NGG1 and NGG2 with pegRNA-guided ABE8e-SpCas9(D10A) adenine base editor (ABE)^66^ to test if a base editor could edit either protospacer. Reassuringly, we observed efficient A•T-to-G•C editing at adenine bases within the expected editing window of ABE8e at NGG1 and NGG2, averaging up to 45% and 29%, respectively (Fig. 1e), demonstrating that NGG1 and NGG2 were indeed accessible by pegRNA-guided Cas effectors. These results suggest that suboptimal execution of the PE steps that follow target site engagement was primarily responsible for inefficient *CFTR* F508del correction.

### PE advances enhance correction of *CFTR* F508del

**Fig. 2:**
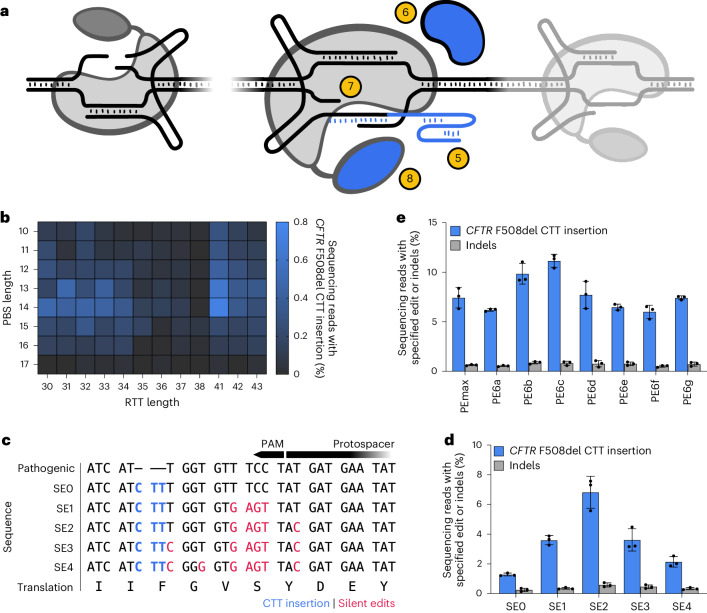
PE enhancements synergistically enhance correction of *CFTR* F508del. **a**, Schematic of the PE system with enhancements that improve F508del correction. Enhancements include (5) epegRNA 3′ structured RNA motifs, (6) co-expression of MLH1dn, (7) translationally silent edits to evade cellular mismatch repair and (8) engineered and evolved prime editor proteins (PEmax and PE6). **b**, Heatmap of F508del correction in HEK293T cells using NGG2 epegRNAs with variable combinations of PBS and RTT lengths, in nucleotides. The edits were completed with PE4max. **c**, Silent edit installation strategies SE0–SE4 are shown. The correction of the F508del CTT deletion alone is shown as SE0. **d**, PE5max correction of F508del in HEK293T cells with the NGG2 PBS13 RTT41 epegRNA is encoded with SE0–SE4. **e**, Comparison of F508del correction with PEmax and PE6 variants a–g in HEK293T cells. All conditions use the NGG2 PBS13 RTT41 SE2 epegRNA, MLH1dn and the +104 ngRNA. For **b**, **d** and **e**, data and error bars represent mean and standard deviation, respectively, of three independent biological replicates (shown as black dots). Source data

Several advances in PE were developed during the course of this study (Fig. 2a). The first of these improvements was the development of engineered pegRNAs (epegRNAs), which protect pegRNA RTT and PBS sequences from endogenous exonuclease degradation by appending an RNA pseudoknot motif to pegRNA 3′ ends^40^. We also reported that inhibition or evasion of cellular DNA MMR enhances PE purity and efficiency^41^ and that PEmax, an architecture-optimized prime editor protein, further increased editing efficiency^41^. Recently, the PE6 suite of laboratory-evolved and engineered RTs and prime editor Cas9 domains with enhanced editing capabilities were reported^34^. Given that these PE enhancements address distinct bottlenecks in the PE mechanism, we sought to combine their capabilities to improve *CFTR* F508del correction.

**Extended Data Fig. 2:**
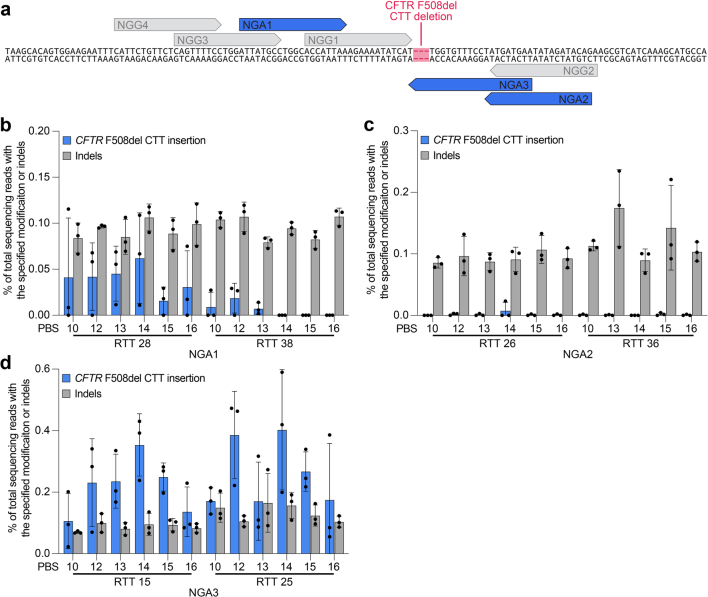
PE4max correction of *CFTR* F508del in HEK293T cells with epegRNAs at several targets with NGA PAMs. **a**, A schematic of the targeted NGA1-3 protospacers around the *CFTR* F508del CTT deletion. **b**–**d**, PE4max *CFTR* F508del correction using epegRNAs with variable PBS lengths and variable RTT lengths targeted to the protospacers NGA1 (**b**), NGA2 (**c**), NGA3 (**d**). PBS and RTT lengths are listed in nucleotides. For (**b**–**d**), data and error bars represent mean and standard deviation, respectively, collected from three independent biological replicates (shown as black dots). Source data

**Extended Data Fig. 3:**
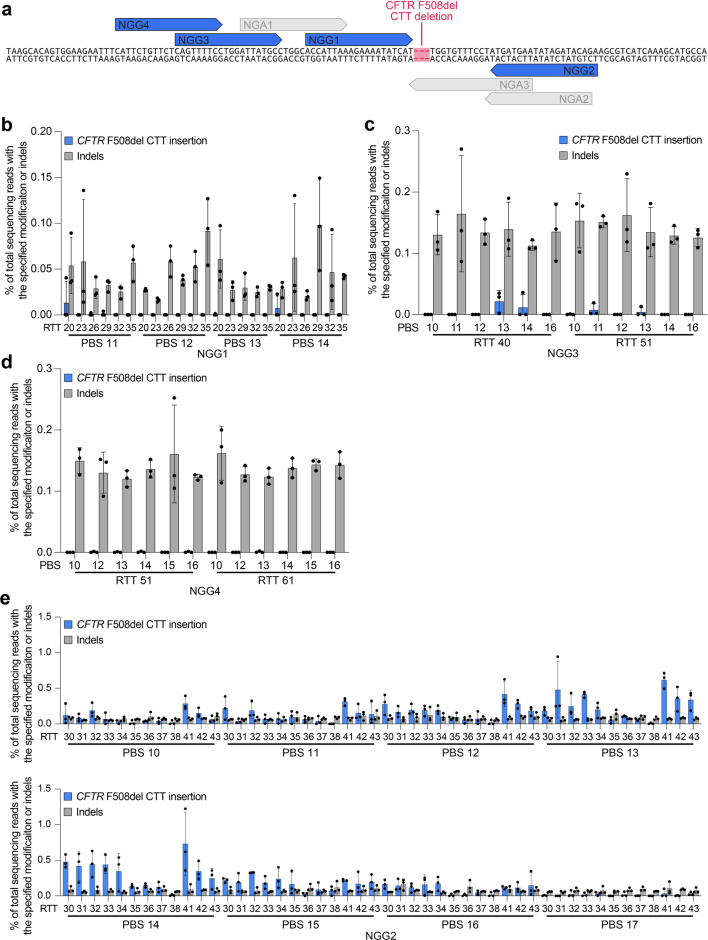
PE4max correction of *CFTR* F508del in HEK293T cells with epegRNAs at several targets with NGG PAMs. **a**, A schematic of the targeted NGG1-4 protospacers around the *CFTR* F508del CTT deletion. **b-e**, PE4max *CFTR* F508del correction using epegRNAs with variable PBS lengths and variable RTT lengths targeted to the protospacers NGG1 (**b**), NGG3 (**c**), NGG4 (**d**), NGG2 (**e**). PBS and RTT lengths are listed in nucleotides. For (**b**–**e**), data and error bars represent mean and standard deviation, respectively, collected from three independent biological replicates (shown as black dots). Source data

We first screened 178 epegRNAs with combinations of PBS and RTT lengths at seven protospacers to explore correction from several F508del-proximal protospacers (Extended Data Figs. 2 and 3). Four protospacers had NGG PAMs (NGG1–NGG4, including NGG1 and NGG2 from our initial PE2 and PE3 experiments) and three protospacers had NGA PAMs (NGA1–NGA3), which have been demonstrated as targetable by prime editors with SpCas9-VRQR(H840A) Cas9 nickase domains^67^. This first screen was completed using PE4max with epegRNAs (where PE4 denotes PE2 + MLH1dn, a dominant-negative MLH1 variant that impedes MMR). We observed marginal rates of editing with the NGA1, NGA2, NGG1, NGG3 and NGG4 epegRNAs (<0.1% F508del correction) but up to 0.40% average correction of F508del with NGA3 (Extended Data Figs. 2b–d and 3b–d). For NGG2, we were motivated by the detectable editing in our earlier PE2 and PE3 experiments to test a wide panel of 96 epegRNAs with different combinations of PBS and RTT lengths (Fig. 2b and Extended Data Fig. 3e). From the NGG2 PE4max panel we identified the epegRNA NGG2 PBS13 RTT41 strategy, which yielded a mean PE4max editing rate of 0.61%, an approximately 3.8-fold improvement over our previous best PE2 pegRNA outcome. Encouraged by the editing efficiency enhancement offered by epegRNAs, PEmax and MLH1dn, we elected to move forward with further F508del edit optimization with the NGG2 PBS13 RTT41 epegRNA.

**Extended Data Fig. 4:**
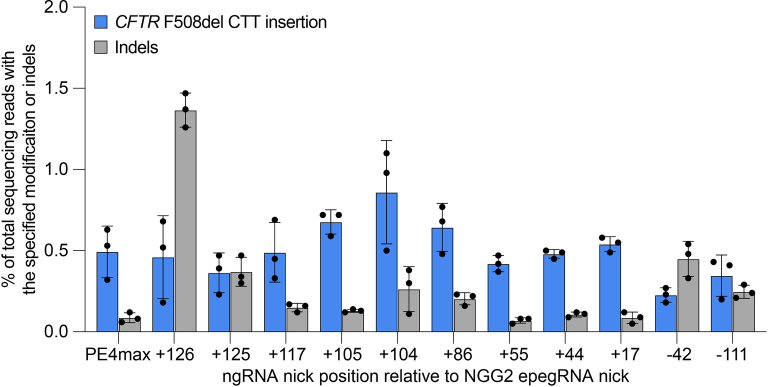
Correction of *CFTR* F508del in HEK293T cells with several ngRNAs using PE5max and an epegRNA. Using the epegRNA NGG2 PBS13 RTT41, a PE5max experiment was performed against a panel of ngRNAs to identify the most efficient strategy to correct *CFTR* F508del. X-axis labels identify different ngRNAs by their nicking position relative to the epegRNA nick (in base pairs). The PE4max x-axis label specifies an editing condition with no ngRNA. Data and error bars represent mean and standard deviation, respectively, collected from three independent biological replicates (shown as black dots). Source data

To further develop the editing strategy to correct F508del, we tested the ngRNA panel from our earlier PE3 experiments with the epegRNA NGG2 PBS13 RTT41 in a PE5max experiment (where PE5 denotes PE3 + MLH1dn) (Extended Data Fig. 4). We identified a +104 nick that enabled a mean editing rate of 0.86% when used with the epegRNA NGG2 PBS13 RTT41, representing a twofold improvement over the previous best PE3 editing strategy and further demonstrating the utility of epegRNAs, PEmax and MLH1dn when paired with a ngRNA.

**Extended Data Fig. 5:**
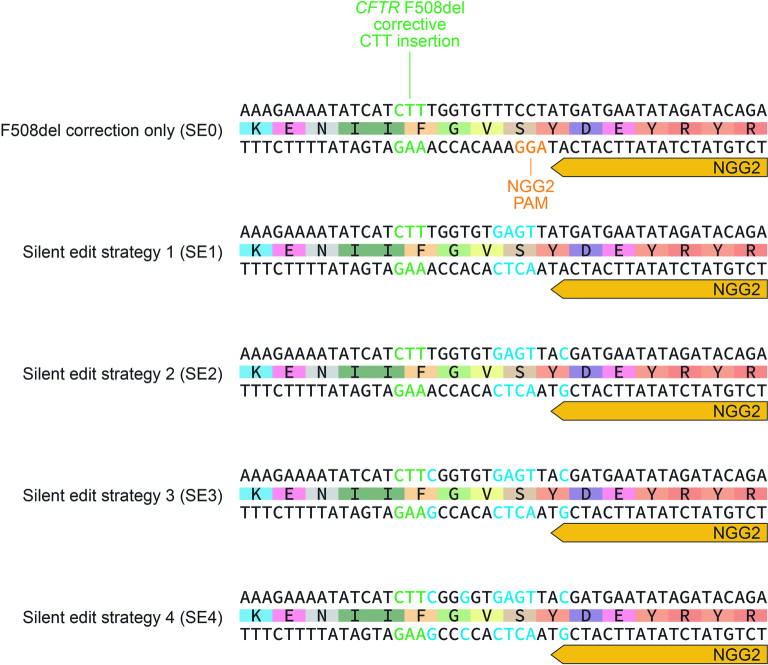
Silent edits co-installed with the F508del corrective CTT insertion disrupt NGG2’s PAM and recode several F508del mutation proximal codons. Schematics of the silent edits (shown as blue base pairs) co-installed with the F508del corrective CTT insertion (shown as green base pairs) as part of silent edit strategies SE1-SE4. The undisrupted PAM of NGG2 is shown as orange base pairs in the SE0 schematic. Source data

Once we identified a suitable epegRNA and ngRNA pair, we sought to further enhance PE by installing benign silent edits simultaneously with the CTT insertion that corrects F508del. We selected silent edits with two purposes: (1) to disrupt NGG2’s PAM and/or protospacer seed sequence, and thereby enhance edit installation efficiency^1^, and (2) to install additional silent edits around the PAM-disrupting edits, thereby creating a contiguous or semi-contiguous tract of mismatches that would cause the PE heteroduplex intermediate to evade MMR, enhancing editing efficiency^41^. In PE5max experiments with the NGG2 PBS13 RTT41 epegRNA, we tested four PE strategies that co-installed silent edits with F508del correction (strategies SE1 to SE4). These strategies took advantage of the flexible third nucleotide position in phenylalanine, glycine, valine and tyrosine codons and the ability of serine codons to be completely recoded (Fig. 2c and Extended Data Fig. 5). Compared with correction of F508del alone without any additional silent mutation installation (SE0), SE2’s co-installation of two NGG2 PAM-disrupting edits and three additional silent mutations resulted in a large (fivefold) improvement in F508del correction, with a mean editing rate of 6.8% (Fig. 2d). This result suggests that additional silent edits to disrupt an epegRNA protospacer’s PAM and enhance MMR evasion can offer major increases in PE efficiency, even when the co-installed silent edits are distal from the corrective edit.

The last PE enhancement that we evaluated in the F508del HEK293T cell line was the use of recently evolved PE6 prime editor protein variants^34^. Using the optimized NGG2 PBS13 RTT41 SE2 epegRNA with MLH1dn and the +104 ngRNA, we compared PEmax with the PE6 variants PE6a–PE6g (Fig. 2e). We observed enhanced F508del correction with PE6b and PE6c compared with PEmax, with mean editing rates of 9.8%, 11% and 7.4%, respectively, consistent with previous observations that PE6b and PE6c enhance editing efficiencies at a variety of targets^34^. Notably, PE6c offered a 1.5-fold improvement in editing activity over PEmax using the NGG2 PBS13 RTT41 SE2 epegRNA, consistent with our previous observations that PE6c can outperform PEmax at many challenging therapeutic edits that use a long RTT^34^. These enhancements of PE efficiencies demonstrate that, when matched with edits tailored to their respective strengths, PE6 variants can synergistically improve PE efficiencies in coordination with other PE improvements including epegRNAs, MLH1dn and silent edits.

### Interdependent PE enhancements in immortalized airway cells

**Extended Data Fig. 6:**
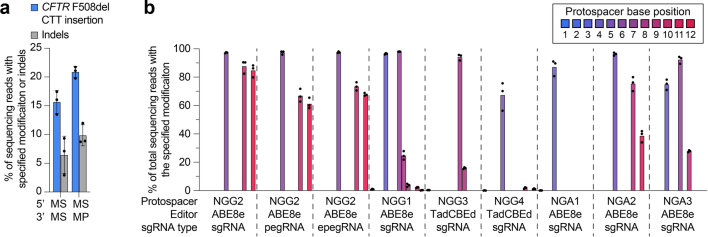
Prime and base editing in 16HBEge-F508del cells via mRNA electroporation. **a**, Effect of epegRNA 3′ modifications on prime editing efficiency at *CFTR* F508del. Both epegRNAs consist of NGG2 PBS13 RTT41 SE2, and both have three phosphorothioate (mS) modifications in the place of phosphodiester bonds on their 5′ end. One epegRNA incorporates 3 mS modifications on its 3′ end and the other incorporates 2 phosphonoacetate (mP) modifications on its 3′ end. **b**, Base editing at *CFTR* F508del proximal protospacers. Deamination efficiencies for all target bases within the first 12 bases of the guide RNA protospacer sequence (indexed from the PAM-distal end of the protospacer) are shown. For (**a**–**b**), data and error bars represent mean and standard deviation, respectively, collected from three independent biological replicates (shown as black dots). Source data

Having identified a suitable PE6-based strategy for the correction of *CFTR* F508del in HEK293T cells, next we tested and further optimized the PE strategy in immortalized human bronchial epithelial cells. We used 16HBE14o- cells homozygous for F508del and M470 (16HBEge-F508del) generated by the Cystic Fibrosis Foundation^68^. These cells show reduced CFTR protein expression and impaired chloride channel function^68^. We delivered the prime editor components identified in the above experiments into 16HBEge-F508del cells as RNA (in vitro-transcribed mRNA for PE and MLH1dn proteins; chemically synthesized RNAs for epegRNA and ngRNA) via electroporation. We found that epegRNAs protected with 3′-phosphonoacetate (mP) modifications enhanced editing efficiency by 1.3-fold over epegRNAs protected with 3′-phosphorothioate modifications, consistent with past studies that found the mP modification to enhance editing by Cas9 nucleases^69^ (Extended Data Fig. 6a). We recommend using synthetic epegRNAs protected with 3′-mP modifications instead of 3′-phosphorothioate modifications to maximize editing efficiency when delivering prime editor components using RNA electroporation.

**Fig. 3:**
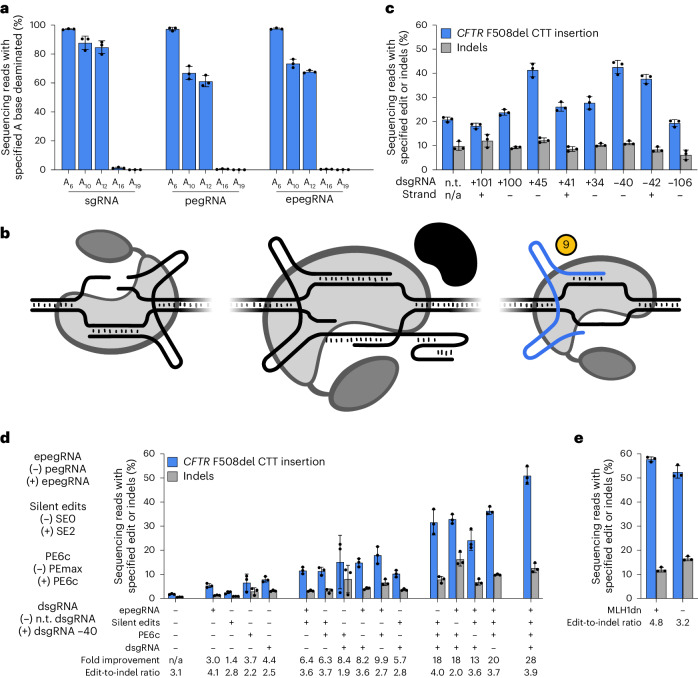
Enhanced PE systems enable F508del correction in human immortalized airway epithelial cells. **a**, ABE of 16HBEge-F508del cells at NGG2 guided by an sgRNA, pegRNA or epegRNA. Adenines (A) are numbered 5′ to 3′ starting from the PAM-distal end of the protospacers. **b**, Schematic of the PE system with dsgRNA (9) added to modulate the chromatin state of the target locus. **c**, Comparison of F508del PE correction in 16HBEge-F508del cells with NGG2-proximal dsgRNAs. dsgRNA *x*-axis label indicates the distance (in nucleotides) between the nicking site of NGG2 and the putative nicking site of the dsgRNA. The strand *x*-axis label indicates the genomic DNA strand to which the dsgRNA binds (NGG2 targets the (–) strand). All conditions use PE6c, the NGG2 PBS13 RTT41 SE2 epegRNA, MLH1dn and the +104 ngRNA. **d**, Combinatorial improvements in F508del correction with epegRNAs, silent edits, PE6c and dsgRNA. For epegRNAs, (–) denotes the use of a pegRNA and (+) denotes the use of an epegRNA; for silent edits, (–) denotes the use of SE0 and (+) denotes the use of SE2; for PE6c, (–) denotes the use of PEmax and (+) denotes the use of PE6c; and for dsgRNA, (–) denotes the use of a non-targeting (n.t.) dsgRNA and (+) denotes the use of the −40 dsgRNA. All conditions use MLH1dn co-expression and the +104 ngRNA. **e**, Effect of MLH1dn co-expression on F508del correction efficiency and edit-to-indel ratio. All conditions use PE6c, the NGG2 PBS13 RTT41 SE2 epegRNA, the −40 dsgRNA and the +104 ngRNA. For **a**–**e**, data and error bars represent mean and standard deviation, respectively, collected from three independent biological replicates (shown as black dots). n/a, not applicable. Source data

*CFTR* is actively transcribed in 16HBEge-F508del cells^68^, suggesting that the chromatin state of the gene may make it more accessible to gene editing agents than it is in HEK293T cells. We nonetheless sought to determine whether chromatin accessibility or prime editor-specific editing constraints may be the limiting factor for F508del correction in 16HBEge-F508del cells. To assess these possibilities, we targeted ABEs as well as cytosine base editors to NGG and NGA protospacers adjacent to F508del using standard Cas9 single-guide RNAs (sgRNAs) and pegRNAs. We observed efficient base editing at all protospacers tested (87–98% editing for ABEs and 67–94% editing for cytosine base editors; Extended Data Fig. 6b). However, we observed that editing rates were lower at bases A_10_ and A_12_ of protospacer NGG2 when ABE was targeted using a pegRNA or an epegRNA compared with an sgRNA (Fig. 3a). This result suggests that although Cas domains can efficiently engage *CFTR* in 16HBEge-F508del cells, the pegRNAs or epegRNAs necessary for PE may hinder the editors’ ability to bind the target site, potentially due to the reduced affinity that Cas9 displays for pegRNAs and epegRNAs compared with sgRNAs^40^ resulting in a smaller fraction of prime editors existing in a guide RNA-bound, target-searching state. This observation led us to hypothesize that we may be able to increase the accessibility of prime editors to *CFTR* by further modulating local chromatin context.

5′-Truncated guide RNAs with only 14–16 nucleotides of protospacer complementarity enable Cas9 to bind to a target sequence but do not support DNA cleavage^70^. We hypothesized that leveraging one such catalytically ‘dead’ sgRNA (dsgRNA) to direct the prime editor protein to a site proximal to F508del would disrupt local chromatin state and increase the accessibility of the prime editor to the site of the therapeutic edit (Fig. 3b); this strategy has been shown to improve PE efficiency in mouse cells^9^. We designed eight NGG-PAM dsgRNAs with 14-nucleotide spacers that bind to sequences within 110 bases of NGG2 and tested their effect on F508del correction when co-delivered with PE6c into 16HBEge-F508del cells. We found that three of the eight tested dsgRNAs substantially improved editing efficiency by an average of 1.9-fold. The best-performing dsgRNA, binding at position −40 relative to the nick location specified by the NGG2 epegRNA, enabled a twofold increase in *CFTR* F508del correction (to a mean efficiency of 42.6%) compared with a control that used a non-targeting dsgRNA (Fig. 3c). We included the −40 dsgRNA in subsequent experiments with 16HBEge-F508del cells and primary CF airway epithelial cells from patients.

Thus, over the course of our efforts to maximize PE-mediated F508del correction in HEK293T and 16HBEge-F508del cell lines, we identified four enhancements—epegRNAs, silent edits, PE6 variants and dsgRNAs—that each substantially increased editing efficiency. Since these enhancements each improve editing efficiency by a different hypothesized mechanism, we tested how the inclusion of each enhancement, both alone and in combination, contributes to efficient editing in 16HBEge-F508del cells. We found that all four enhancements are essential for maximizing editing efficiency in 16HBEge-F508del cells (Fig. 3d). In the absence of these four key enhancements, PE5max F508del correction averaged 1.8% with 0.57% indel formation. With all four enhancements implemented, we observed a mean F508del correction rate of 51% with 13% indel formation and a 28-fold improvement in editing efficiency, while also increasing the edit-to-indel ratio from 3.1 to 3.9. In a separate experiment, we sought to characterize the influence of MLH1dn on editing efficiency in the presence of these enhancements. We found that omitting MLH1dn led to a modest reduction in F508del correction efficiency from 58% to 52% and a decrease in edit-to-indel ratio from 4.8 to 3.2 (Fig. 3e). These findings indicate that mechanistically distinct improvements to PE systems contribute synergistically to enhanced editing efficiencies.

### Reevaluation of the *CFTR* F508del PE correction strategy

**Extended Data Fig. 7:**
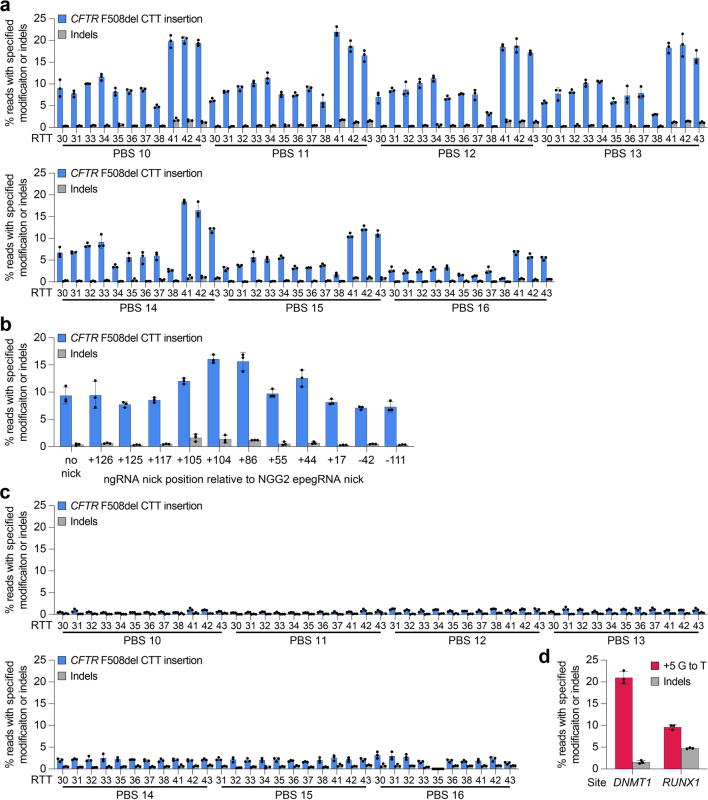
pegRNA, ngRNA, and petRNA optimization in HEK293T cells in the context of other prime editing enhancements. **a**, epegRNA PBS and RTT length screen using SE2 silent edits, PE6c, MLH1dn, the +104 ngRNA, and the -40 dsgRNA. **b**, ngRNA screen using PE6c, MLH1dn, epegRNA NGG2 PBS13 RTT41 SE2, and the -40 dsgRNA. **c**, petRNA screen using SE2 silent edits, PE6c, MLH1dn, the +104 ngRNA, and the -40 dsgRNA. **d**, Editing efficiencies using petRNAs at previously reported positive control target sites. For (**c**) and (**d**), a nCas9 (based on PEmax) and a MCP-RT (using a PE6c-based reverse transcriptase) were used for petRNA editing, as previously described^71^. For (**a**–**d**), data and error bars represent mean and standard deviation, respectively, collected from three independent biological replicates (shown as black dots). Source data

Given the inefficient editing rates we observed in our initial pegRNA optimization efforts using PE2 and PE4max (Figs. 1b and 2b), we sought to determine whether the epegRNA characteristics we selected remained optimal in the presence of other enhancements. We rescreened epegRNA PBS and RTT lengths at NGG2 in *CFTR* F508del HEK293T cells using PE6c with MLH1dn, SE2 silent edits, the −40 dsgRNA and the +104 ngRNA and found that PBS13 RTT41 was still among the most optimal PBS and RTT combinations (Extended Data Fig. 7a). Rescreening nicking sgRNAs also indicated that the +104 nsgRNA remained optimal (Extended Data Fig. 7b). These results suggest that optimal epegRNA and ngRNA designs for the *CFTR* F508del corrective edit remain the same in the context of diverse PE enhancements, and therefore, for at least some target sites, these characteristics can be optimized independently of other improvements to the PE system.

We considered that the marginal PE2 and PE4max editing rates (Figs. 1b and 2b) may be the result of epegRNA PBS–RTT instability or *cis*-acting interactions between the spacer and the PBS. To circumvent these potential issues, we tested the prime editing template RNA (petRNA) system in which epegRNAs are split into separate sgRNAs and circularized RTT–PBS transcripts^71^. Using combinations of an NGG2 sgRNA and petRNAs with varied PBS and RTT lengths, we rescreened F508del correction in *CFTR* F508del HEK293T cells using MLH1dn, SE2 silent edits, the −40 dsgRNA and the +104 ngRNA (Extended Data Fig. 7c). As previously described^71^, an nCas9 (here based on PEmax) and an MS2 coat protein (MCP)–RT (here using a PE6c-based RT) were used for petRNA editing. While we were able to recapitulate efficient petRNA editing at *DNMT1* and *RUNX1* positive control targets (Extended Data Fig. 7d), we did not observe *CFTR* F508del correction greater than 4%, indicating that the use of petRNAs did not enhance PE correction of F508del.

**Extended Data Fig. 8:**
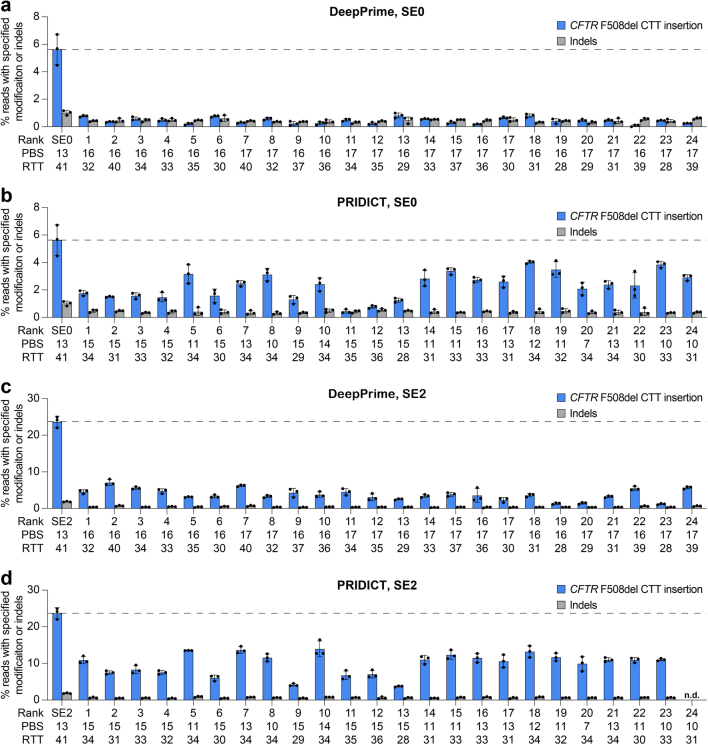
*CFTR* F508del correction efficiency in HEK293T cells by epegRNAs designed using computational tools. *CFTR* F508del correction mediated by the top 24 ranked epegRNAs designed by: **a**, DeepPrime without the co-installation of silent edits (SE0); **b**, PRIDICT without the co-installation of silent edits (SE0); **c**, DeepPrime with the co-installation of SE2 silent edits; and **d**, PRIDICT with the co-installation of SE2 silent edits. All conditions use PE6c, MLH1dn, the +104 ngRNA, and the -40 dsgRNA. For (**a**–**d**) data and error bars represent mean and standard deviation, respectively, collected from three independent biological replicates (shown as black dots). n.d., no data; this epegRNA was not cloned due to DNA synthesis errors. Source data

We also sought to assess whether epegRNA screening could be accelerated using computational tools for pegRNA design. Using PE6c with MLH1dn, the −40 dsgRNA and the +104 ngRNA, we tested the F508del correction efficiencies of the top 24 epegRNAs predicted by PRIDICT^72^ and the top 24 epegRNAs predicted by DeepPrime^73^. However, neither model predicted NGG2 PBS13 RTT41 within their top 24 designs; this epegRNA outperformed all others generated from the suggestions of both models in the context of both SE0 and SE2 (Extended Data Fig. 8). On the basis of these results, we recommend experimental epegRNA screening to identify epegRNAs that yield the highest editing efficiencies, rather than relying solely on current computational models.

### PE restores CFTR function in primary airway epithelial cells

**Fig. 4:**
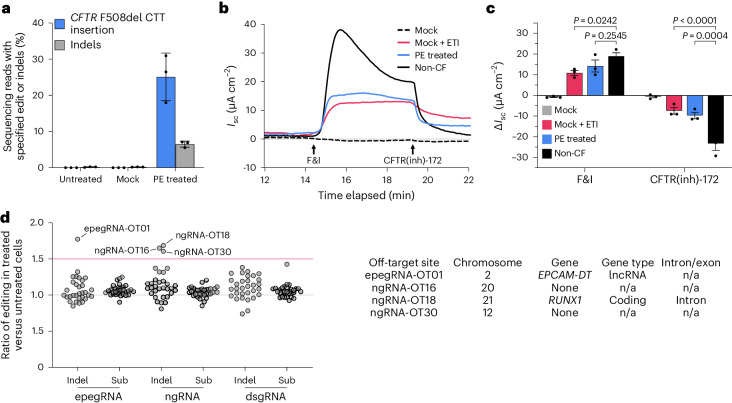
PE corrects *CFTR* F508del in primary airway epithelial cells from patients with CF and rescues ion channel function. **a**, Quantification of F508del correction in *CFTR* F508del homozygous primary airway epithelial cells from patients with CF using HTS. The untreated condition indicates primary cells that were not electroporated. Data and error bars represent mean and standard deviation, respectively, and were collected from three independent donors (shown as black dots). **b**, The representative Isc recordings are shown with the transepithelial voltage held at 0 mV. A total of 3 weeks after electroporation of PE reagents, the transepithelial *I*_sc_ of fully differentiated airway epithelial cells was quantified in response to F&I and CFTR(inh)-172 treatment. All the cells were pre-incubated with 10 µM forskolin and 100 µM IBMX for 24 h before recording Cl^−^ transport. Non-CF cultures and donor-matched F508/F508 cultures with ETI pretreatment served as positive controls. **c**, Summary of the change in short-circuit current (Δ*I*_sc_) in response to F&I and CFTR(inh)-172 treatment. Data and error bars represent mean and standard error of the mean, respectively, and were collected from three independent donors (shown as black dots). *P* values, determined by two-way ANOVA, are shown. For **a**–**c**, mock condition indicates primary cells that were electroporated without RNA. **d**, Ratio of editing in treated versus untreated primary airway epithelial cells at off-target loci nominated by CIRCLE-seq. Indels and substitutions (Sub) are shown for sites cleaved in vitro by the epegRNA, ngRNA and dsgRNA used in our PE strategy. For the epegRNA and dsgRNA, the top 31 CIRCLE-seq nominated off-target sites are shown as one of the top 32 identified sites was *CFTR* F508del. For the ngRNA, the top 32 CIRCLE-seq nominated off-target sites are shown. All data points are the ratios of the mean of three replicates for treated and untreated samples. The red line indicates a ratio of editing in treated versus untreated cells of 1.5. The characteristics of off-target sites for which this ratio exceeds 1.5 are shown in the table to the right. Observed editing frequency is the mean of three independently edited primary airway epithelial cell lines. lncRNA, long non-coding RNA; n/a, not available. Source data

Encouraged by the development, optimization and characterization of an efficient PE *CFTR* F508del correction strategy in 16HBEge-F508del cells, we next tested our F508del correction strategy in primary human airway epithelial cells from three patients with homozygous *CFTR* F508del alleles. Using PE6c with MLH1dn, the −40 dsgRNA, the +104 ngRNA and the NGG2 PBS13 RTT41 SE2 epegRNA delivered by RNA electroporation, we observed a mean F508del correction rate of 25% with 6.5% indels (Fig. 4a) among all treated cells with no sorting.

To determine the phenotypic impact of correcting F508del in primary CF airway epithelial cells, we quantified CFTR-mediated anion secretion in prime-edited donor cells under short-circuit conditions following differentiation for 3 weeks at an air–liquid interface (ALI). The change in short-circuit current (*I*_sc_) was recorded following stimulation with the CFTR-activating agents forskolin and 3-isobutyl-1-methylxanthine (IBMX; ‘forskolin and IBMX’ is abbreviated as F&I) and inhibition with the CFTR-specific channel inhibitor CFTR(inh)-172. Donor-matched mock-treated epithelial cells (electroporated without RNA), epithelial cells treated with elexacaftor, tezacaftor and ivacaftor (ETI) and non-CF epithelial cells were included as electrophysiological controls. Compared with mock-treated CF donor epithelial cells, F&I stimulation resulted in substantially increased CFTR-dependent Isc in the prime editor-treated cells and CFTR(inh)-172 administration likewise decreased the CFTR-dependent Isc in the prime editor-treated cells (Fig. 4b,c). Compared with treatment with ETI, prime editor-treated cells exhibited a similar change in CFTR-dependent Isc when stimulated with F&I or after CFTR(inh)-172 administration (Fig. 4b,c). These results demonstrate substantial rescue of CFTR channel activity following correction by PE, with CFTR function in prime editor-treated donor cells exceeding 50% of current levels in healthy (non-CF) airway epithelial cells (Fig. 4c).

To assess the persistence of F508del correction during cellular differentiation, we evaluated F508del correction rates in airway epithelial cells from two ALI-cultured populations. We found that rates of F508del correction remained consistent when compared at 3 days post-electroporation and after 3 weeks of differentiation at ALI (Supplementary Fig. 3), demonstrating the edit’s stability during cellular expansion. These findings demonstrate that the optimized PE strategy not only restores expression of functional CFTR capable of much-improved anion transport in primary CF airway epithelial cells from patients, but also that the corrective edit persists through cellular proliferation and differentiation.

### On-target and off-target editing analysis

**Extended Data Fig. 9:**
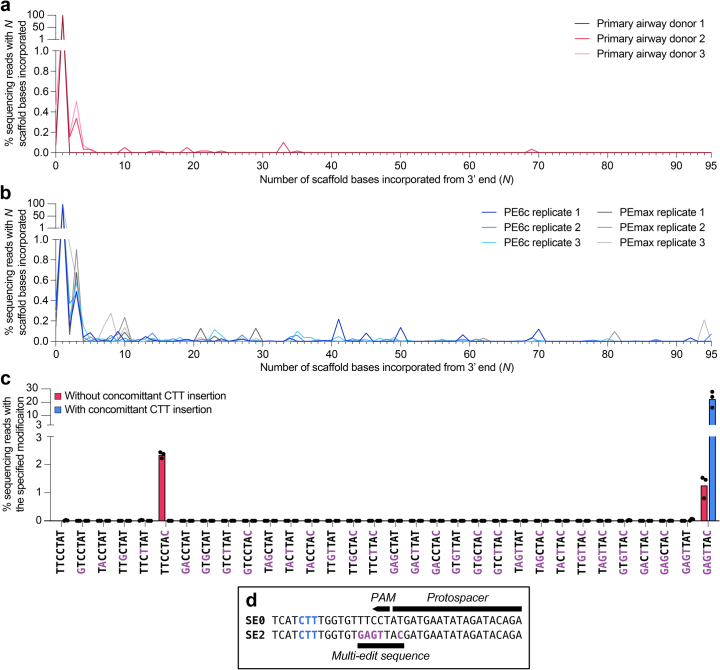
Scaffold integration and partial edit incorporation in prime edited cells. **a**, Scaffold integration in prime edited primary airway epithelial cells from patients with CF, edited in Fig. 4a. The frequency of HTS reads containing CTT insertion and a defined number of scaffold-incorporated bases, indexed from the 3′ end of the pegRNA scaffold, is shown. **b**, Scaffold integration in prime edited 16HBEge-F508del cells. Scaffold integration frequencies are shown as described in (**a**) for both PEmax and PE6c edited cells. Data collected from Fig. 3d condition (+) epegRNA, (+) SE2, (- and +) PE6c, and (+) dsgRNA -40. **c**, Occurrences of all possible combinations of SE2 edit incorporation, both with and without concomitant CTT insertion, in PE-treated primary airway epithelial cells from Fig. 4a. **d**, Schematic showing the location of Cas9 PAM and protospacer edits within SE2. For (**a**–**c**), the epegRNA used consists of NGG2 PBS13 RTT41 SE2 with 2 × 3′ phosphonoacetate modifications. Source data

Having established that enhanced PE systems can efficiently correct *CFTR* F508del and restore CFTR function in primary CF airway epithelial cells, we sought to characterize unintended editing outcomes of our PE strategy—including epegRNA scaffold integration, partial silent edit incorporation and genomic off-target editing—in detail. The highly processive Tf1 RT used in PE6c can generate indels by reverse transcribing the epegRNA scaffold, which can be incorporated into the target genomic DNA^34^. To determine if this scaffold incorporation occurs in prime-edited primary CF airway epithelial cells, we analysed high-throughput sequencing (HTS) reads for partial and complete scaffold integration events. The first base of the epegRNA scaffold, indexed from the 3′ end, is complementary to the first base of genomic DNA encoded beyond the RTT; we therefore cannot distinguish between 0 and 1 bases of scaffold integration, as these lead to identical (scaffold-free) sequence outcomes. Across three edited donor cell lines, we observed no scaffold incorporation in 98.8–99.9% (mean 99.2%) of reads containing F508del CTT insertion (Extended Data Fig. 9a). We also observed no additional scaffold incorporation in the three replicates of 16HBEge-F508del cells edited with high-processivity PE6c (percentage of CTT insertion events that are scaffold-free: 97.9–98.0%, mean 98.0%) compared with PEmax (percentage of CTT insertion events that are scaffold-free: 97.3–98.7%, mean 98.0%) (Extended Data Fig. 9b). These findings are consistent with the previous observation that highly processive PE6 variants do not increase rates of scaffold incorporation when carefully matched with edits suited to their capabilities (such as using PE6c to enhance editing with the long 3′ extension of the NGG2 PBS13 RTT41 SE2 epegRNA)^34^. As predicted by NUPACK^74^, the folding energy of the PBS and RTT of the NGG2 PBS13 RTT41 SE2 epegRNA is −7.80 kcal mol^−1^. The choice of PE6c for this edit is consistent with our prior findings that Tf1-based PE variants (PE6b and PE6c) frequently outperform alternatives for epegRNAs with predicted secondary structure folding energies smaller in magnitude (less stable) than −23 kcal mol^−1^ (ref. ^34^).

The *CFTR* F508del correction strategy developed above installs silent edits—most notably, converting a serine codon from TCC to AGT—to evade cellular MMR mechanisms. Partial incorporation of these silent edits could lead to non-synonymous changes in protein sequence. We therefore searched for the occurrence of all possible combinations of SE2 partial silent edit incorporation, both with and without concomitant CTT insertion, in treated primary airway epithelial cells. We found that partial silent edits are incorporated at a low frequency without CTT insertion, and we did not observe the incorporation of any partial edits that would lead to outcomes encoding non-synonymous protein sequences (Extended Data Fig. 9c,d). Importantly, some of these silent edits also disrupt Cas9 protospacer and PAM sequences and thus should prevent Cas9 re-engagement after editing. We observed the incorporation of protospacer and PAM edits without concomitant CTT insertion in primary CF airway epithelial cells with a mean frequency of 3.6% (compared with a mean CTT insertion frequency of 23%). This editing outcome effectively renders a small fraction of F508del-containing sequences un-targetable by PE, eliminating their ability to be corrected. The increase in CTT insertion frequency we observe when installing silent edits therefore greatly outweighs the fraction of un-correctable sequences generated due to partial silent edit incorporation. We recommend that partial silent edit incorporation be investigated in future therapeutic PE studies, especially if some silent edits encode changes to the protospacer or PAM sequences.

**Extended Data Fig. 10:**
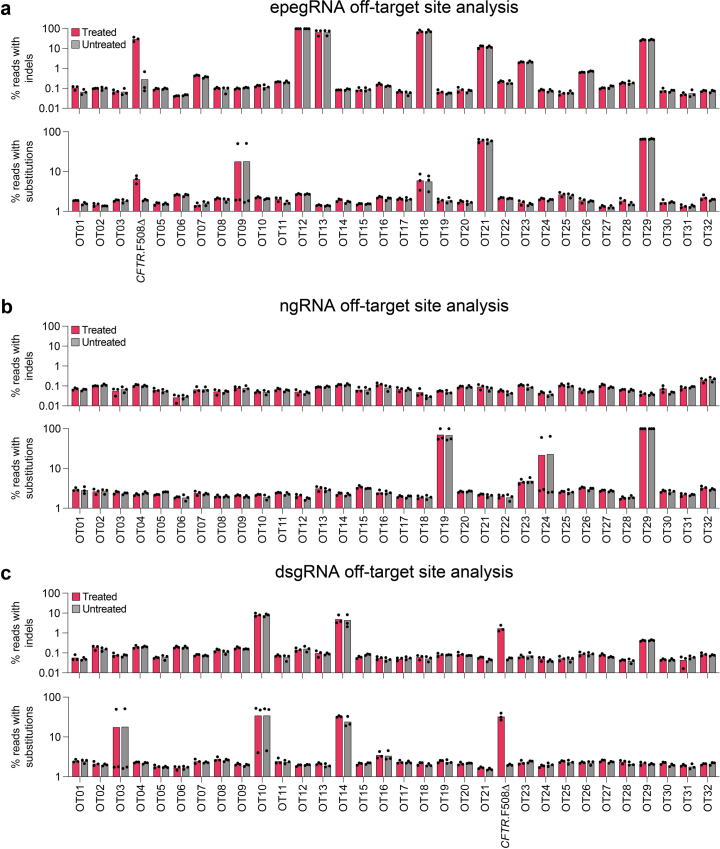
Analysis of off-target editing in primary airway epithelial cells from patients with CF. **a**, Indel and substitution quantification at the top 32 human genomic sites identified by CIRCLE-seq for epegRNA NGG2 PBS13 RTT41 SE2. **b**, Indel and substitution quantification at the top 32 human genomic sites identified by CIRCLE-seq for ngRNA +104. **c**, Indel and substitution quantification at the top 32 human genomic sites identified by CIRCLE-seq for dsgRNA –40. For (**a**–**c**) data represents mean collected from three independent biological replicates (shown as black dots). Source data

To characterize off-target editing frequencies of the optimized PE strategy, we used CIRCLE-seq^75^ to identify sites within the human genome that are cleaved in vitro by Cas9 guided with the epegRNA, ngRNA or dsgRNA used in our *CFTR* F508del PE strategy. We assessed editing outcomes in primary CF airway epithelial cells from three separate donors at the top 32 candidate sites for each of the three guide RNAs, representing 96 total CIRCLE-seq-nominated off-target candidate sites investigated (Supplementary Tables 3–5). We observed few differences in off-target indel or substitution formation in PE-treated donor cells compared with untreated donor cells (Fig. 4d and Extended Data Fig. 10a–c), consistent with the observation from multiple laboratories that PE induces few detected off-target modifications, probably due to its mechanism requiring multiple DNA hybridization events^1,3,5–17^. Of the 96 total CIRCLE-seq nominated off-target sites we assessed in primary CF airway epithelial cells, 4 showed a mean 1.5-fold or higher increase in indel formation in the treated cells compared with the untreated cells (Fig. 4d), potentially suggestive of off-target editing. No off-target sites showed a mean 1.5-fold or higher increase in substitution formation in treated cells compared with untreated cells.

The indel frequencies at all four of these potential off-target sites were ≤0.1% in treated samples across all replicates, indicating that the off-target modification at these sites occurs at a very low frequency. We nonetheless analysed these sites more closely to assess their potential physiological relevance (Fig. 4d). One of the four sites is engaged by the epegRNA (epegRNA-OT01) and targets the *EPCAM-DT* long non-coding RNA, which has no known function. The remaining three sites are engaged by the ngRNA. Two of these sites lie within intergenic regions and are unlikely to have strong physiological consequences if disrupted at a low frequency. The final site (ngRNA-OT18) is situated within an intronic region of *RUNX1*^76^. Because ngRNA-OT18 is located ~60 kb from the nearest exon and showed a low mean indel formation rate of 0.048% in treated primary cells, we do not anticipate this off-target site to carry substantial physiological consequences. No potential off-target sites were detected from the dsgRNA, which may be explained by the inability of Cas9 nuclease to cleave DNA targets when guided with a 6-bp truncated guide sequence^70^. These observations collectively suggest that the PE *CFTR* F508del correction strategy induces few off-target edits in the human genome under the tested conditions.

## Discussion

By systematically refining a PE strategy to correct *CFTR* F508del, we achieved large improvements in editing efficiency. Our initial PE3 experiments only resulted in up to 0.42% mean F508del correction. By applying several recently described PE improvements to the *CFTR* F508del correction strategy, we achieved up to 11%, 58% and 25% mean F508del correction in HEK293T cells, 16HBEge-F508del cells and primary airway epithelial cells from patients with CF, representing 26-fold, 140-fold and 59-fold improvements in F508del correction over the original PE3 editing attempt, respectively. Six improvements to the original PE system each contributed to the optimization of our F508del correction strategy: (1) epegRNAs, (2) PEmax, (3) MLH1dn, (4) silent edits, (5) PE6 and (6) dsgRNAs. Experiments in F508del 16HBEge-F508del cells demonstrated that, when used in combination, epegRNAs, silent edits, PE6c and dsgRNAs can each contribute comparably to a 28-fold net improvement in editing efficiency (Fig. 3d). Consistent with the inherent resistance of the PE mechanism to off-target editing, we observed only very low frequencies (≤0.1% at four sites) of potential off-target editing in primary airway epithelial cells from patients with CF following treatment with the optimized PE correction system. Previous reports have noted the difficulty of PE correction of *CFTR* F508del^8^ and other CF-causing mutations in *CFTR*^8,77^. We hope the systematic process we used to achieve efficient F508del correction starting from very low initial editing efficiency can serve as a guide to optimize other therapeutic PE efforts.

During this study, we evaluated PE advances in the order determined by the chronological development of each technology. As such, our initial epegRNA and ngRNA screens yielded only low rates of F508del correction (Fig. 2b and Extended Data Fig. 4, respectively). A limitation of these early experiments was their narrow dynamic range of editing efficiencies, which made certain design choices—such as the selection of PBS and RTT lengths—somewhat subjective. Results from the retrospective epegRNA and ngRNA screens with all PE enhancements (Extended Data Fig. 7a,b) generally confirmed that our design choices were among the most optimal. If all PE advances had been available during our initial epegRNA and ngRNA screens, we would have used a PE6 editor or silent edits to broaden the dynamic range of our editing and enhance the confidence of our design decisions. For future research facing low initial PE efficiencies, we suggest adopting this approach.

We observed variable rates of F508del correction in our HEK293T, 16HBEge and primary airway cell experiments. These different rates of PE F508del correction may be due to transfection efficiency, transfection method-related toxicity and, for primary cells, donor genetic variability or the preservation status of primary donor tissues. For example, we observed near 100% editing in 16HBEge cell ABE8e experiments (Fig. 3a), indicating near 100% transfection efficiency of 16HBEge cells, while fluorescence-activated cell sorting of *CFTR* F508del HEK293T cells transfected with a PE6c-P2A-EGFP (where EGFP is enhanced green fluorescent protein) construct revealed only 19% transfection efficiency (Supplementary Figs. 4a and 5), resulting in bulk and GFP-enriched F508del mean correction rates of 9% and 35%, respectively (Supplementary Fig. 4b). The comparable mean F508del correction rates of 35% and 58% between GFP-enriched HEK293T and 16HBEge cells, respectively, suggest that transfection efficiency variability among unsorted cell populations could account for the observed variability in F508del correction rates among different cell types.

In CF donor airway epithelial cells, the 25% mean F508del correction rescued CFTR ion channel function to greater than 50% of healthy non-CF airway epithelial cells. Previously, similar ranges of in vitro F508del mutation correction and functional CFTR rescue were reported with Cas9 nuclease-mediated homology-directed repair (HDR) of upper airway basal stem cells and airway epithelial cells^78^. However, while the HDR-mediated editing strategy used Cas9 nuclease to induce DSBs and adeno-associated virus type 6 to provide a donor DNA template, our PE F508del correction strategy is DNA-free and takes advantage of transient RNA reagents that do not require DSBs. The DSB-independent nature of PE may offer fewer undesired and uncharacterized outcomes and obviate the need for donor DNA template delivery, simplifying delivery and reducing cellular toxicity. Notably, the HDR strategy relied on the use of Rho-kinase inhibitor Y-27632 to improve cellular proliferation following editor treatment^78^, presumably to overcome toxicity, whereas our approach does not require the addition of small molecules to enhance cell survival.

Although our PE strategy generates indels at substantially lower frequencies than nuclease-mediated strategies, these indel byproducts should still be evaluated for potential adverse effects in patients. Despite yielding similar levels of F508del correction, the previously reported HDR-mediated editing strategy resulted in a nearly 1:1 edit-to-indel ratio^78^, whereas our PE-mediated F508del correction method offers a mean edit-to-indel ratio of 3.8 in primary cells. Crucially, the enhanced edit-to-indel ratio of the PE strategies developed in this study, compared with the use of Cas9 nuclease, results in unedited alleles predominantly encoding the unmodified F508del CFTR protein that remains druggable by CF small-molecule correctors and potentiators, unlike the frameshifted *CFTR* alleles from the much higher frequency of nuclease-mediated indel byproduct formation that probably generate truncated CFTR proteins that cannot be rescued by small-molecule drugs. Additionally, frameshifted alleles may generate neoantigens that could provoke an immune response in patients. However, because frameshifted alleles lead to a non-functional CFTR protein, we would not expect immune clearance of these cells to affect the therapeutic efficacy of our editing strategy. Minimizing immune reactivity by minimizing indel formation via strategies such as PE over nuclease-mediated HDR may nonetheless increase overall editing efficiency in vivo. Ex vivo editing and expansion of correctly edited cells followed by engraftment using electroporation-based techniques, such as those developed for the treatment of CF upper airway pathologies^78^, could also overcome indel-related safety issues. As we demonstrate in both 16HBEges and primary cells, our all-RNA PE system is amenable to delivery via electroporation.

Our PE strategy to correct *CFTR* F508del benefits from recent advances in precision gene editing, offering distinct advantages over nuclease-mediated HDR editing. However, previous efforts to correct *CFTR* F508del with HDR have provided valuable insights and technical developments that contextualize our PE results and suggest potential applications^78^. HDR correction of *CFTR* F508del achieved gene editing rates similar to our PE approach and led to the finding that such levels of F508del correction would be clinically beneficial if replicated in vivo, as supported by several in vitro studies^79–81^ and clinical analyses of milder CF genotypes^82–85^. We can extend this finding to our observations following PE-mediated F508del correction, given the similar levels of functional CFTR rescue. Overall, our work complements previous studies on HDR-mediated correction of *CFTR* F508del and introduces a state-of-the-art approach that achieves high correction-to-indel ratios without the need for DSBs, DNA delivery, or small-molecule inhibitors, substantially simplifying potential clinical applications.

A critical challenge to realizing the therapeutic potential of our PE strategy for *CFTR* F508del correction will be the development of technologies to deliver our composition of matter to relevant airway tissues in vivo. Several key PE enhancements—including the use of an epegRNA, a dsgRNA and MLH1dn co-expression—involve nucleic acid or protein components in addition to the core PE system. We find that each of these enhancements plays a role in maximizing F508del mutation correction in disease-relevant cell lines (Fig. 3d). Strategies for the efficient in vivo delivery of the optimized F508del PE correction system developed in this work, which totals ~8.5 kb of sequence without promoters or regulatory elements, to the lung epithelium are thus crucial for realizing the potential of PE to correct cystic fibrosis-causing mutations. Numerous potentially feasible delivery modalities suited for large gene editing cargoes have been reported. These include helper-dependent adenoviral vectors (HDAd)^86,87^, multiple adeno-associated virus^5,29,32–38^, engineered virus-like particles^88^, lipid nanoparticles with lung tissue tropism that have been leveraged to deliver Cas9 mRNA^89,90^ and engineered amphiphilic peptides that have been used to deliver Cas9 ribonucleoproteins and base editors to airway epithelia^91,92^. Several humanized *CFTR* murine models have been recently developed^93,94^ that could serve as suitable models for the development and optimization of in vivo F508del PE delivery strategies.

HDAd vectors are a particularly attractive strategy due to their large packaging limits (~37 kb)^86^, their demonstrated ability to deliver cargoes to mouse and pig airway basal epithelia in vivo^87^ and their ability to efficiently package and deliver PE5max to human cells^31^. Importantly, a recent study that delivered PE5max via HDAd to a mouse model of sickle cell disease found no detrimental effects associated with MLH1dn co-expression compared with PE3max^31^. This suggests that transient delivery of MLH1dn, which is crucial for maximizing edit-to-indel ratio in our editing strategy (Fig. 3e), may be a viable therapeutic option. However, given the potential side-effects of MLH1 inactivation, the magnitude and duration of MLH1dn co-expression should be minimized when optimizing in vivo delivery strategies. The modest reduction in editing efficiency we observe when omitting MLH1dn from our editing strategy in the presence of other enhancements such as silent edits that also serve to avoid the consequences of MMR suggests that MLH1dn could be avoided without substantially sacrificing therapeutic efficacy. One therapeutic technique that would circumvent the need for in vivo delivery of prime editor components is to harvest and edit regenerative airway basal cells ex vivo^95^. Although ex vivo editing would probably enable higher editing efficiencies than in vivo delivery, ex vivo correction has numerous limitations that may limit its therapeutic efficiency, including the need to expand short-lived basal cells following editing to obtain sufficient numbers for engraftment^96^ and competition between endogenous airway cells and donor cells that diminishes engraftment efficiency^97^.

The PE optimization approach in this study could also be used to develop precise editing strategies for class I mutations that produce no functional CFTR protein and therefore do not benefit from small-molecule correctors and potentiators^45^. The recently reported addition of the RNA-binding protein SSB/La^98^, as well as future enhancements, may enable further increases in the correction efficiency of CF-causing mutations. Additionally, the recent use of PE and recombinases to install gene-sized (>5 kb) cargoes into target genomic sites^99–101^ could provide a mutation-agnostic approach to the genetic correction of CF. Because CF is an autosomal recessive disorder, one could envision using PE-mediated recombination following a similar optimization process to what we describe here to install a functional copy of *CFTR* into the native site or into a genomic safe harbour locus to treat CF.

## Methods

### General molecular biology

Guide RNA expression plasmids (pegRNAs, epegRNAs, ngRNAs, dsgRNAs and sgRNAs) were cloned as previously described using isothermal assembly and synthetic gene fragments^61^. Guide RNA sequences are provided in Supplementary Table 2. Guide RNA expression plasmids were purified using PureYield Plasmid Miniprep kits (Promega). Prime editor and MLH1dn plasmids were purified using Qiagen Plasmid Plus Midi kit (Qiagen). DNA PCR amplification was completed using Phusion U Green Multiplex PCR master mix (Thermo Fisher Scientific). Primers and gene fragments were ordered from Integrated DNA Technologies. New prime editor plasmids were cloned using isothermal assembly, and sequences are provided in Supplementary Information.

### Synthetic guide RNA generation

Synthetic pegRNAs and epegRNAs were ordered from Agilent Research Labs and contained 2′-O-methyl modifications at the first three nucleotides, 2′-O-methyl modifications at the third-to-last and second-to-last nucleotides, 3′-phosphorothioate linkages between the first three nucleotides and 3′-mP linkages between the last two nucleotides. Synthetic ngRNAs were ordered from Synthego and contained 2′-O-methyl modifications at the first three and last three nucleotides and 3′-mP linkages between the first three and last two nucleotides. Synthetic dsgRNAs were ordered from Integrated DNA Technologies and contained 2′-O-methyl modifications at the first three and last three nucleotides and phosphorothioate linkages between the three first and last three nucleotides. Guide RNA sequences are provided in Supplementary Table 2.

### Generation of in vitro-transcribed mRNAs

The prime editor, ABE, cytosine base editor and MLH1dn-encoded mRNA were generated using in vitro transcription as previously described^61^. Briefly, the prime editor, base editor or MLH1dn transcripts, containing a 5′ untranslated region, Kozak sequence, prime editor or MLH1dn open reading frame, and 3′ untranslated region were PCR amplified from a template plasmid containing an inactive T7 (dT7) promoter. PCR primers repaired this dT7 promoter and also installed a 119 nt poly(A) tail. The purified PCR double-stranded DNA amplicon was used as an in vitro transcription template using the HiScribe T7 high-yield RNA synthesis kit (NEB). In vitro transcription followed the manufacturer’s optional protocol to include CleanCap reagent AG (Trilink) and substitute N1-methylpseudouridine-5′-triphosphate (Trilink) for uridine triphosphate. Lithium chloride precipitation was used to purify mRNA from complete in vitro transcription reactions, and mRNA transcripts were reconstituted in nuclease-free water.

### General mammalian cell culture conditions

HEK293T (ATCC CRL-3216) cells were purchased from American Type Culture Collection and cultured in Dulbecco’s modified Eagle medium with GlutaMax (Thermo Fisher Scientific) supplemented with 10% foetal bovine serum (Thermo Fisher Scientific) at 37 °C with 5% CO_2_, as previously described^61^.

16HBEge-F508del cells homozygous for *CFTR* F508del were a gift from the Cystic Fibrosis Foundation Therapeutics Lab and were cultured as previously described^68^. Briefly, 16HBEge-F508del cells were cultured in minimum essential medium (Thermo Fisher Scientific) supplemented with 10% foetal bovine serum (Thermo Fisher Scientific) and 1× penicillin–streptomycin (Thermo Fisher Scientific) at 37 °C with 5% CO_2_. For routine cell culture, standard tissue culture flasks were pre-treated with a thin layer of coating solution at 37 °C 5% CO_2_ for 2 h, followed by coating solution removal and coated flask storage at 4 °C. Coating solution composition was LHC-8 basal medium (Thermo Fisher Scientific) supplemented with 1.34 µl ml^−1^ bovine serum albumin 7.5% (Thermo Fisher Scientific), 10 μl ml^−1^ bovine collagen solution type 1 (Advanced BioMatrix) and 10 µl ml^−1^ fibronectin from human plasma (Thermo Fisher Scientific).

Primary airway epithelial cells from non-CF donors were isolated from trachea or bronchi from post-mortem lungs unsuitable for transplant. Primary CF airway epithelial cells were isolated from lung tissue obtained following lung transplant. Studies were approved by the University of Iowa Institutional Review Board, under the US Department of Health and Human Services registration number IRB00000099. The genotypes of the CF donors were F508del/F508del (*n* = 3 donors). Cells were expanded on culture plates coated with human collagen IV (Sigma) in PneumaCult-Ex Plus medium (STEMCELL Technologies). To establish ALI cultures, cells were seeded onto collagen-coated, semi-permeable membranes (0.33 cm^2^, polycarbonate, Costar) and differentiated in PneumaCult-ALI medium (STEMCELL Technologies) for ≥3 weeks before functional assays.

All cell lines were verified to be free of mycoplasma and identity-authenticated by their suppliers.

### HEK293T transfection

HEK293T transfections were conducted as previously described^61^. Briefly, approximately 16,000 HEK293T cells were plated per well on a 96-well plate in complete culture media. After 18–24 h, cells were transfected at 70–80% confluency with variable amounts of plasmid DNA and 0.5 µl of Lipofectamine 2000 diluted in Opti-MEM (Thermo Fisher Scientific), following the manufacturer’s instructions.

For a single 96-well transfection using standard prime editor systems, the following plasmids amounts were transfected: 200 ng of prime editor; 50 ng of pegRNA, epegRNA or sgRNA; 15 ng of ngRNA (if included in experiment); 15 ng of dsgRNA (if included in experiment); and 100 ng of MLH1dn (if included in experiment).

For a single 96-well transfection using the petRNA system with a split prime editor^71^, the following plasmid amounts were transfected: 100 ng of the PEmax nCas9, 100 ng of the MCP–RT(PE6c), 25 ng of petRNA, 25 ng of petRNA-paired sgRNA, 15 ng of ngRNA, 15 ng of dsgRNA and 100 ng of MLH1dn.

Following transfection, cells were incubated at 37 °C with 5% CO2 for 72 h.

### 16HBEge-F508del cell and primary airway epithelial cell electroporation

Electroporation of 16HBEge-F508del cells and primary airway epithelial cells from patients with CF was completed with the SG Cell Line 4D-Nucleofector X Kit (Lonza) and a 4D-Nucleofector (Lonza). Cells from maintenance culture were dissociated using TrypLE (Thermo Fisher Scientific), and 200,000 cells were resuspended in 20 µl complete SG nucleofector solution per reaction. PE RNA reagents were added the nucleofector solution cell mix as follows: 1 µg prime editor mRNA, 1 µg MLH1dn mRNA, 90 pmol pegRNA or epegRNA, 30 pmol ngRNA, and 30 pmol dsgRNA. The cell–RNA mixture was transferred into a nucleofector 16-well strip and electroporated using the programme CM-137. Following electroporation, 80 µl of pre-warmed growth media was added to each cuvette and the 100 µl cell mix was used to seed tissue culture plates.

For 16HBEge-F508del cells, cells were transferred onto a 24-well tissue culture plate with pre-warmed 16HBEge-F508del cell growth media and incubated at 37 °C in 5% CO_2_ for 6 days. On day 4, cells were rinsed with phosphate-buffered saline to eliminate dead cells and subsequently incubated with fresh growth media.

For primary CF airway epithelial cells, cells were transferred onto collagen-coated six-well plates with pre-warmed Ex Plus medium and incubated at 37 °C in 5% CO_2_. Cells were rinsed with phosphate-buffered saline to eliminate dead cells and subsequently incubated with fresh Ex Plus medium the following day. Seventy-two hours after electroporation, cells were dissociated with TrypLE and seeded onto collagen-coated semi-permeable Transwell membranes at density of 1.25 × 10^5^ per filter in Ex Plus medium. Cells were maintained in Ex Plus medium for 2–4 days and subsequently switched to ALI medium. The cells were differentiated at ALI for 3 weeks before functional analysis. A cell aliquot obtained during filter seeding was used for genomic DNA extraction and HTS analysis.

### Genomic DNA preparation from cell culture

Genomic DNA (gDNA) was isolated from HEK293T and 16HBEge-F508del cell culture following a custom lysis protocol and paramagnetic bead extraction. To lyse cells, growth medium was carefully removed from cell culture plates and 100 µl or 250 µl of lysis buffer (100 mM Tris-HCl pH 8.0, 200 mM NaCl, 5 mM EDTA, 0.05% SDS, 4.0 mg ml^−1^ proteinase K (New England Biolabs), and 12.5 mM dithiothreitol) was added to each well of a 96-well or 24-well plate, respectively. Plates with lysis buffer were incubated for 20 h at 55 °C with shaking. To extract gDNA, one volume of lysate was thoroughly mixed with one volume of Ampure XP beads (Beckman Coulter Life Sciences) and incubated for 5 min, separated on plate magnet, washed with 70% ethanol three times with bead resuspension and eluted in 50 µl nuclease-free water.

Genomic DNA from primary CF airway epithelial cells was extracted using QuickExtract (LGC Biosearch Technologies) lysis solution, following the manufacturer’s protocol.

### Preparation of HEK293T cells for fluorescence-activated cell sorting

Briefly, for a single confluent well of HEK293T cells in a 96-well plate: 72 h after transfection, medium was carefully removed and 30 µl TrypLE Express (Thermo Fisher Scientific) was used to coat the cell monolayer, which was then incubated at 37 °C with 5% CO2 for 5 min. Following incubation, 70 µl complete HEK293T cell growth medium was added and cells were resuspended by pipetting. The cell–medium mix was centrifuged at 250*g* for 5 min, medium supernatant was removed and pelleted cells were resuspended in 500 µl phosphate-buffered saline (Thermo Fisher Scientific), and the cell–medium mix was passed through a 35-μm cell strainer (Corning). Cells were sorted using a MA900 Cell Sorter (Sony Biotechnology) at the Broad Institute Flow Cytometry Core. See Supplementary Fig. 5 for the fluorescence-activated cell sorting gating strategy. Cells were sorted into custom HEK293T cell lysis buffer (listed above), and gDNA was extracted as described for the custom lysis buffer (procedure listed above).

### HTS and data analysis

HTS of genomic loci was completed as previously described^61^. All HTS primers are provided in Supplementary Table 1. Briefly, two rounds of PCR amplification were performed using Phusion U Green Multiplex PCR master mix (Thermo Fisher Scientific). In the first round of PCR amplification, approximately 150 ng of gDNA was used to template a PCR with primers containing Illumina adaptor overhangs (Supplementary Table 1) and cycled under the following conditions: 95 °C for 3 min, 27–30 cycles of 95 °C for 10 s, 58–61 °C (corresponding to the experimentally optimized Tm) for 20 s and 72 °C for 30 s, followed by 72 °C for 5 min. In the second round of PCR, combinations of Illumina-barcoded forward and reverse primers were used to uniquely identify each sample and, with 1–2 µL of PCR round 1 as a template, were amplified under the following conditions: 95 °C for 3 min, seven cycles of 95 °C for 10 s, 61 °C for 20 s and 72 °C for 30 s, followed by 72 °C for 5 min. PCR round 2 reactions from the same genomic locus were pooled and gel extracted with QIAquick gel extraction kit (Qiagen) to eliminate primer dimers. Gel-extracted amplicons were quantified using Qubit double-stranded DNA high-sensitivity assay kit (Thermo Fisher Scientific), and 4 nM libraries were run on an Illumina MiSeq 300 v2 Kit with 200–300 cycles.

Sequencing reads were de-multiplexed using MiSeq Reporter (Illumina) and amplicon sequences were aligned to a reference sequence using CRISPResso2^102^, as previously described^61^. For PE experiments, the parameter --quantification_window_coordinates (QWC) was set as the amplicon coordinates 10 bp 5′ upstream from the nick position of the most 5′ epegRNA, pegRNA, ngRNA or dsgRNA and 10 bp 3′ downstream from the nick position of the most 3′ epegRNA, pegRNA, ngRNA or dsgRNA. If the end of the 3′ flap generated by the RT plus 10 bp extended past one of these defined QWC bounds, the amplicon coordinate of the 3′ flap generated by the RT plus 10 bp was used in place of the superseded QWC bound. For all CRISPResso2 runs the discard_indel_reads parameter was set as ‘TRUE’, the *q* parameter set as ‘30’ and, for base editing experiments, the parameter *w* was used in place of QWC and set to ‘10’. Additionally, in all CRISPResso2 runs, indels for a given sample were calculated as ((‘Discarded’/‘Reads_aligned_all_amplicons’) × 100) using data from the ‘CRISPRessoBatch_quantification_of_editing_frequency.txt’ output file. For single-base changes, editing efficiency at a given position was calculated as ((frequency of specified point mutation from the ‘Nucleotide_percentage_summary.txt’ file) × (‘Reads aligned’/‘Reads_aligned_all_amplicons’) × 100), where the ‘Reads aligned’ and ‘Reads_aligned_all_amplicons’ values were collected from the ‘CRISPRessoBatch_quantification_of_editing_frequency.txt’ output file. For precise PE insertion or deletion edits, CRISPResso2 was run in HDR mode and editing efficiency was calculated as ((HDR amplicon ‘Reads_aligned’/‘Reads_aligned_all_amplicons’) × 100) using the ‘CRISPRessoBatch_quantification_of_editing_frequency.txt’ output file.

### Generation of a homozygous *CFTR* F508del HEK293T cell line using PE2

The monoclonal *CFTR* F508del HEK293T cell line was isolated by limiting dilution. Briefly, after identifying the most efficient PE2 strategy to install the *CFTR* F508del CTT deletion into the endogenous *CFTR* gene of HEK293T cells (Supplementary Fig. 1), HEK293T cells were transfected with this PE2 strategy following the methods listed above. After 72 h, transfected cells were dissociated from adherent culture, counted and plated across ten 96-well plates in 100 µl of medium per well at a concentration of 0.5 cells per each well. Cells were grown for 10 days and monitored for the development of monoclonal cell populations. Identified monoclonal cultures were expanded and genotyped following the HTS methods listed above. One clonal HEK293T cell line homozygous for *CFTR* F508del was isolated.

### Design of epegRNAs using computational tools

Web servers for DeepPrime (https://deepcrispr.info/DeepPrime/) and PRIDICT 2.0 (https://pridict.it) were used to design pegRNA sequences for CTT insertion at *CFTR* F508del. Default settings were used for both models. The desired edited sequence used as a model input was simple CTT insertion without concomitant silent edits (that is, SE0). The 24 pegRNA sequences with the highest predicted editing efficiency were synthesized as epegRNAs and tested experimentally. SE2 epegRNAs were designed by incorporating the SE2 silent edits into the SE0 pegRNA sequences generated by each model.

### Ussing chamber assay

A total of 3 weeks following electroporation, primary airway epithelial cell cultures were treated for 24 h with 10 µM forskolin (Cayman) and 100 µM IBMX (Sigma) before bioelectric studies. Donor-matched naive CF cultures treated for 24 h with ETI (2 μM of elexacaftor (VX-445, Cayman), 18 μM of tezacaftor (VX-661, Cayman) and 1 μM of ivacaftor (VX-770, Cayman) in the presence of 10 mg ml^−1^ normal human serum (Sigma), as described previously^54^, were used as a control for CFTR modulator treatment. Non-CF cultures treated with 10 µM forskolin and 100 µM IBMX were used as non-CF positive control.

Epithelial cell cultures were mounted in Ussing chambers (Physiologic Instruments) in symmetrical Cl^−^-buffered Ringer’s solutions consisting of (mM): 135 NaCl, 5 HEPES, 0.6 KH_2_PO_4_, 2.4 K_2_HPO_4_, 1.2 MgCl_2_, 1.2 CaCl_2_ and 5 dextrose, with pH titrated to 7.40 at 37 °C with NaOH. The command voltage was set to 0 mV and *I*_sc_ was monitored. The following drugs were used during the assay: apical amiloride (Sigma-Aldrich, 100 μM from 100 mM DMSO stock) to inhibit ENaC, apical 4, 4’-dilsothiocyano-2, 2’-stilbenedifulonic acid (DIDS, Sigma-Aldrich, 100 μM from 100 mM DMSO stock) to inhibit non-CFTR Cl^–^ channels, apical forskolin (Cayman Chemical, 10 μM from 10 mM DMSO stock) and IBMX (100 μM from 100 mM ethanol stock) to stimulate CFTR channel activity, and CFTR_inh_-172 (Sigma-Aldrich, 10 µM from 10 mM DMSO stock) to block CFTR.

### Prediction of pegRNA secondary structure

NUPACK^74^ (https://old.nupack.org/) was used to predict the secondary structure and folding energies of pegRNAs. The pegRNA RTT and PBS (for epegRNA NGG2 PBS13 RTT41 SE2: UGGCACCAUUAAAGAAAAUAUCAUCUUUGGUGUGAGUUACGAUGAAUAUAGAUA) sequence was queried using default parameters (type: RNA, temperature: 37 °C, maximum complex size: one strand).

### Quantification of pegRNA scaffold insertion

Quantification of pegRNA scaffold insertion was completed using a custom Python script as described previously^1^. The script searches for occurrences of sequences of the pegRNA scaffold adjacent to on-target edits within HTS data, beginning with a single base at the 3′ end of the scaffold (proximal to the pegRNA RTT, referenced in the analysis as base 1). The script iterates through the entire scaffold sequence; in its *i*th iteration, insertion events representing integration of scaffold sequences 1 − *i* are quantified. Scaffold insertion was quantified using genomic DNA from three prime-edited primary CF airway epithelial cell cultures as well as prime-edited 16HBEge-F508del cells. The script used to quantify scaffold integration is available in Supplementary Note 1.

### Analysis of partial edit incorporation

Partial edit incorporation was quantified using CRISPResso2 in HDR mode, as described above (see ‘HTS and data analysis’ section). For each sample, a separate CRISPResso2 run was executed for every possible combination of partial SE2 edit incorporation, both with and without concomitant CTT insertion. Partial edit frequency was calculated as ((HDR amplicon ‘Reads_aligned’/‘Reads_aligned_all_amplicons’) × 100) using the ‘CRISPRessoBatch_quantification_of_editing_frequency.txt’ output file. Partial edit quantification was performed using genomic DNA from three prime-edited primary CF airway epithelial cell cultures as well as three unedited controls.

### Off-target analysis

Off-target site nomination was performed using CIRCLE-seq as previously described^103^. The top 32 CIRCLE-seq nominated off-target sites, as defined by nuclease read count, for each of the three guide RNAs used to edit primary CF airway epithelial cells (epegRNA NGG2 PBS13 RTT41 SE2, ngRNA +104 and dsgRNA −40) were selected for deep sequencing analysis. The nominated sites are listed in Supplementary Tables 3–5. The primers were designed to amplify each of the 96 nominated off-target sites within a 240–280 bp amplicon using National Center for Biotechnology Information Primer-BLAST (Supplementary Table 1). Off-target sites were amplified from purified gDNA and prepared for HTS as described above (see ‘HTS and data analysis’ section). Off-target amplicon sequencing was performed using gDNA from three prime-edited primary CF airway epithelial cell cultures as well as three unedited controls.

Indels and substitutions at nominated off-target sites were quantified from HTS data using CRISPResso2^102^. Indels were quantified within a window from 10 bp 5′ upstream of the end of the epegRNA homology template to 10 bp 3′ downstream of the Cas9 nick position; this was achieved by setting the parameter WC to ‘18’ and the parameter *W* to ‘31’. Substitutions were quantified within a window from 0 bp 5′ upstream of the end of the epegRNA homology template to 0 bp 3′ downstream of the Cas9 nick position; this was achieved by setting the parameter WC to ‘18’ and the parameter *W* to ‘21’. For both indel and substitution quantifications, the discard_indel_reads parameter was set as ‘TRUE’ and the *q* parameter was set as ‘30’. Indels were calculated as ((‘Discarded’/‘Reads_aligned_all_amplicons’) × 100) using data from the ‘CRISPRessoBatch_quantification_of_editing_frequency.txt’ output file from the indel CRISPResso2 run. Substitutions were calculated as ((‘Substitutions’/‘Reads_aligned_all_amplicons’) × 100) using data from the ‘CRISPRessoBatch_quantification_of_editing_frequency.txt’ output file from the substitution CRISPResso2 run. Off-target sites were further investigated if the mean indel or substitution rate at the site for prime-edited samples was 1.5-or-more-fold greater than mean indel or substitution rate for the same site in untreated samples.

### Reporting summary

Further information on research design is available in the Nature Portfolio Reporting Summary linked to this article.

## Supplementary information

Supplementary Information Supplementary figures, notes and sequences.

Reporting Summary

Supplementary Data Supplementary tables of sequencing primers; pegRNA, epegRNA, petRNA, ngRNA, dsgRNA and sgRNA sequences used in each figure and associated Sequence Read Archive file names; and CIRCLE-seq nominated off-target loci.

## Source data

Source Data Figs. 1–4 and Extended Data Figs. 1–10 Source data.

## Data Availability

All data supporting the results of this study are available within the paper and its Supplementary Information. High-throughput sequencing data are available from the NCBI Sequence Read Archive database (PRJNA1055086). Key plasmids are available from Addgene (depositor: D.R.L.), or from the corresponding author on request. Source data are provided with this paper.

## Code availability

The code used for the analysis of the high-throughput sequencing data is available via GitHub at https://github.com/pinellolab/CRISPResso2. The assessment of epegRNA scaffold insertion was completed using a custom Python script described previously^1^ and provided as Supplementary Information.
