# Supplementary material for: Systematic optimization of prime editing for the efficient functional correction of CFTR F508del in human airway epithelial cells
Source: Nat Biomed Eng. 2024 Jul 10;9(1):7–21. doi: 10.1038/s41551-024-01233-3 (PMC11754097; doi:10.1038/s41551-024-01233-3)
Supplement: Supplementary file 1 — Supplementary figures, notes and sequences. [file 41551_2024_1233_MOESM1_ESM.pdf]

nature biomedical engineering

Supplementary information

https://doi.org/10.1038/s41551-024-01233-3

Systematic optimization of prime editing for the efficient functional correction of *CFTR* F508del in human airway epithelial cells

In the format provided by the authors and unedited

Contents

Supplementary Figure 1. Optimization of PE2 strategy to install the *CFTR* F508del mutation in HEK293T cells.

Supplementary Figure 2. *CFTR* F508del proximal protospacers NGG1 and NGG2

Supplementary Figure 3. Editing rates in primary airway cells from patients with CF at three days post-electroporation and following three weeks of differentiation at an air-liquid interface.

Supplementary Figure 4. Evaluation of PE6c transfection efficiency in HEK293T cells.

Supplementary Figure 5. FACS gating strategy for HEK293T cells.

Supplementary Sequences. Sequences of new plasmids used in this study.

Supplementary Note 1. Python script used for quantification of scaffold incorporation.

Supplementary Table 1. Sequencing primers.

(Sequences of primers for genomic amplification are provided as a separate Excel file).

Supplementary Table 2. Sequences of pegRNAs, epegRNAs, petRNAs, ngRNAs, dsgRNAs and sgRNAs used in each figure and associated Sequence Read Archive (SRA) file name.

(Sequences and SRA file names are provided as a separate Excel file).

Supplementary Table 3. CIRCLE-seq nominated off-target loci for epegRNA NGG2 PBS13 RTT41 SE2.

(Provided as a separate Excel file).

Supplementary Table 4. CIRCLE-seq nominated off-target loci for ngRNA +104.

(Provided as a separate Excel file).

Supplementary Table 5. CIRCLE-seq nominated off-target loci for dsgRNA -40.

(Provided as a separate Excel file).

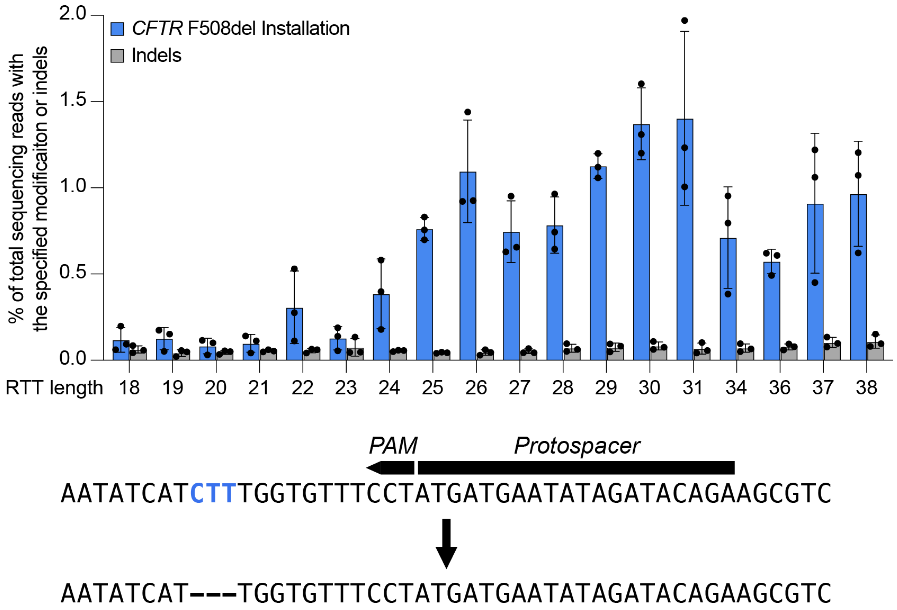

Supplementary Fig. 1 | Optimization of PE2 strategy to install the *CFTR* F508del mutation in HEK293T cells. To identify a PE2 strategy to install the *CFTR* F508del CTT deletion into HEK293T cells (bottom sequence schematic), a panel of 18 pegRNAs with a PBS length of 14 nt and variable RTT lengths was transfected into HEK293T cells (top data plot). One of the most efficient pegRNA designs (PBS14 and RTT30) was selected to generate a monoclonal HEK293T cell line homozygous for the *CFTR* F508del mutation. RTT lengths are listed in nucleotides. Data and error bars represent mean and standard deviation, respectively, collected from three independent biological replicates (shown as black dots).

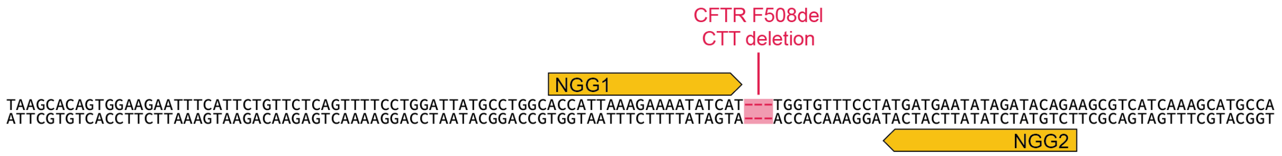

Supplementary Fig. 2 | *CFTR* F508del proximal protospacers NGG1 and NGG2. A schematic of the targeted NGG1 and NGG2 protospacers around the *CFTR* F508del CTT deletion. The non-PAM containing strand of NGG1 contains a TTTT sequence that may act as an RNA Polymerase III transcriptional terminator that prevents complete pegRNA PBS transcription from a U6 promoter.

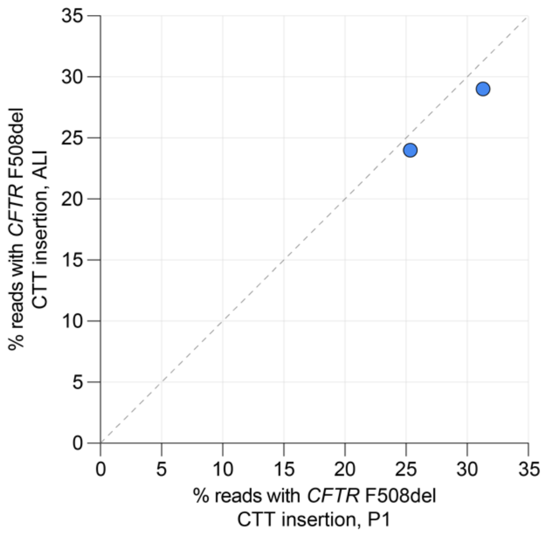

Supplementary Fig. 3 | Editing rates in primary airway cells from patients with CF at three days post-electroporation (P1, x-axis) and following three weeks of differentiation at an air-liquid interface (ALI, y-axis). Each data point represents F508del correction rates for cells from the same donor, quantified via high-throughput sequencing.

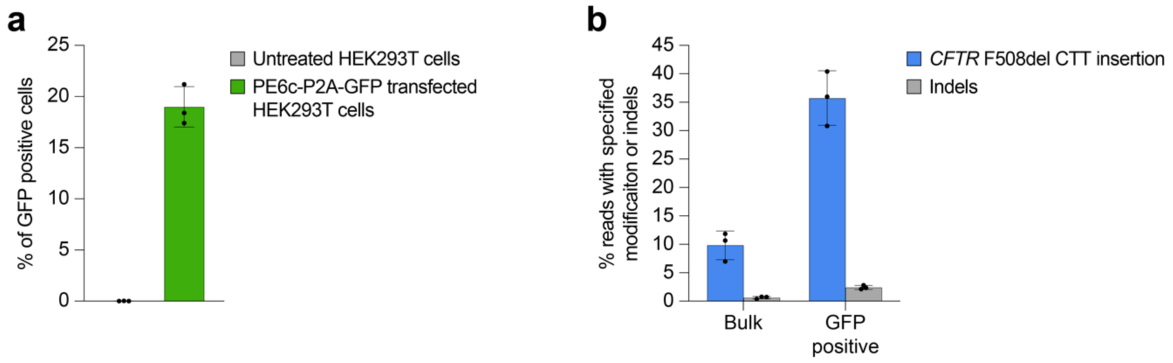

Supplementary Fig. 4 | Evaluation of PE6c transfection efficiency in HEK293T cells. a, Percent GFP positivity of HEK293T cells transfected with PE6c-P2A-GFP, MLH1dn, epegRNA NGG2 PBS13 RTT41 SE2, the +104 ngRNA and the -40 dsgRNA. GFP positivity was assessed through fluorescence-activated cell sorting. **b**, *CFTR* F508del correction efficiency in bulk and GFP-positive cells treated with PE6c-P2A-GFP, MLH1dn, epegRNA NGG2 PBS13 RTT41 SE2, the +104 ngRNA and the -40 dsgRNA. For (**a** and **b**), data and error bars represent mean and standard deviation, respectively, collected from three independent biological replicates (shown as black dots).

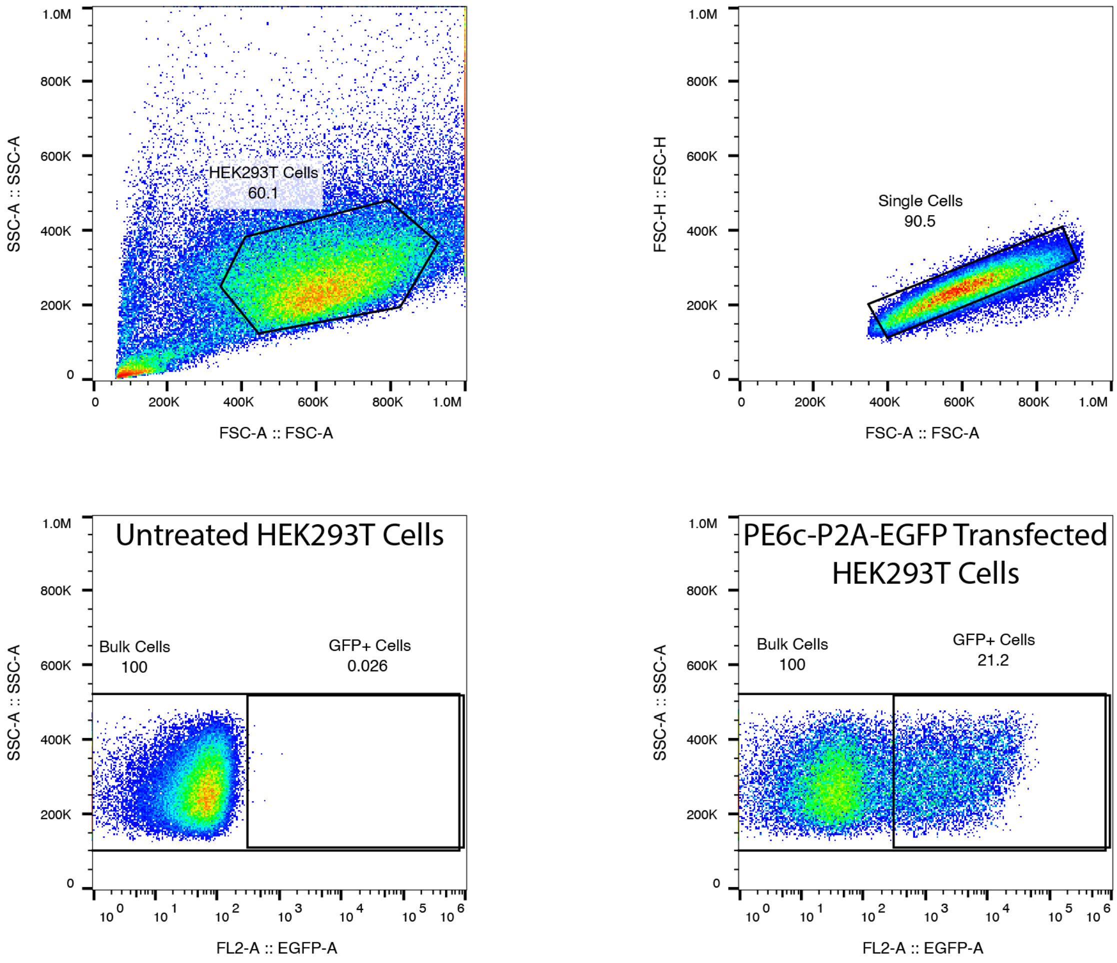

Supplementary Fig. 5 | FACS gating strategy for GPF-positive HEK293T cells. HEK293T cells were harvested from confluent monolayers 72 hours after transfection. Cells were disassociated with trypsin, pelleted, and resuspended in PBS. Gates were drawn around HEK293T cells based on forward and side scatter area, gated on singlets based on FSC-H and FSC-A plots, and then sorted based on GFP fluorescence into a “bulk” population containing all HEK293T cells regardless of GFP positivity, and a GFP-positive (GFP+) population.

Supplementary Sequences. Sequences of new plasmids used in this study.

Sequence of nCas9 used for petRNA experiments

nCas9 created from PEmax[1] construct. The M-MLV reverse transcriptase was removed.

CMV enhancer-CMV promoter-T7 promoter- BPNLS-SV40 NLS-Cas9(R221K N394K H840A)- BPNLS-SV40 NLS-GSG-cmycNLS

GACATTGATTATTGACTAGTTATTAATAGTAATCAATTACGGGGTCATTAGTTCATAGCCCATATATGGAGTTCCGCGTTACATAACTTA
CGGTAAATGGCCCGCCTGGCTGACCGCCCAACGACCCCCGCCCATTGACGTCAATAATGACGTATGTTCCCATAGTAACGCCAATAGGGA
CTTTCCATTGACGTCAATGGGTGGAGTATTTACGGTAAACTGCCCACTTGGCAGTACATCAAGTGTATCATATGCCAAGTACGCCCCCTA
TTGACGTCAATGACGGTAAATGGCCCGCCTGGCATTATGCCCAGTACATGACCTTATGGGACTTTCCTACTTGGCAGTACATCTACGTAT
TAGTCATCGCTATTACCATGGTGATGCGGTTTTGGCAGTACATCAATGGGCGTGGATAGCGGTTTGACTCACGGGGATTTCCAAGTCTCC
ACCCCATTGACGTCAATGGGAGTTTGTTTTGGCACCAAAATCAACGGGACTTTCCAAAATGTCGTAACAACTCCGCCCCATTGACGCAAA
TGGGCGGTAGGCGTGTACGGTGGGAGGTCTATATAAGCAGAGCTGGTTTAGTGAACCGTCAGATCCGCTAGAGATCCGCGGCCGCTAATA
CGACTCACTATAGGGAGAGCCGCCACCATGAAACGGACAGCCGACGGAAGCGAGTTCGAGTCACCAAAGAAGAAGCGGAAAGTCGACAAG
AAGTACAGCATCGGCCTGGACATCGGCACCAACTCTGTGGGCTGGGCCGTGATCACCGACGAGTACAAGGTGCCCAGCAAGAAATTCAAG
GTGCTGGGCAACACCGACCGGCACAGCATCAAGAAGAACCTGATCGGAGCCCTGCTGTTCGACAGCGGCGAAACAGCCGAGGCCACCCGG
CTGAAGAGAACCGCCAGAAGAAGATACACCAGACGGAAGAACCGGATCTGCTATCTGCAAGAGATCTTCAGCAACGAGATGGCCAAGGTG
GACGACAGCTTCTTCCACAGACTGGAAGAGTCCTTCCTGGTGGAAGAGGATAAGAAGCACGAGCGGCACCCCATCTTCGGCAACATCGTG
GACGAGGTGGCCTACCACGAGAAGTACCCCACCATCTACCACCTGAGAAAGAAACTGGTGGACAGCACCGACAAGGCCGACCTGCGGCTG
ATCTATCTGGCCCTGGCCCACATGATCAAGTTCCGGGGCCACTTCCTGATCGAGGGCGACCTGAACCCCGACAACAGCGACGTGGACAAG
CTGTTCATCCAGCTGGTGCAGACCTACAACCAGCTGTTCGAGGAAAACCCCATCAACGCCAGCGGCGTGGACGCCAAGGCCATCCTGTCT
GCCAGACTGAGCAAGAGCAGAAAGCTGGAAAATCTGATCGCCCAGCTGCCCGGCGAGAAGAAGAATGGCCTGTTCGGAAACCTGATTGCC
CTGAGCCTGGGCCTGACCCCCAACTTCAAGAGCAACTTCGACCTGGCCGAGGATGCCAAACTGCAGCTGAGCAAGGACACCTACGACGAC
GACCTGGACAACCTGCTGGCCCAGATCGGCGACCAGTACGCCGACCTGTTTCTGGCCGCCAAGAACCTGTCCGACGCCATCCTGCTGAGC
GACATCCTGAGAGTGAACACCGAGATCACCAAGGCCCCCCTGAGCGCCTCTATGATCAAGAGATACGACGAGCACCACCAGGACCTGACC
CTGCTGAAAGCTCTCGTGCGGCAGCAGCTGCCTGAGAAGTACAAAGAGATTTTCTTCGACCAGAGCAAGAACGGCTACGCCGGCTACATT
GACGGCGGAGCCAGCCAGGAAGAGTTCTACAAGTTCATCAAGCCCATCCTGGAAAAGATGGACGGCACCGAGGAACTGCTCGTGAAGCTG
AAGAGAGAGGACCTGCTGCGGAAGCAGCGGACCTTCGACAACGGCAGCATCCCCCACCAGATCCACCTGGGAGAGCTGCACGCCATTCTG
CGGCGGCAGGAAGATTTTTACCCATTCCTGAAGGACAACCGGGAAAAGATCGAGAAGATCCTGACCTTCCGCATCCCCTACTACGTGGGC
CCTCTGGCCAGGGGAAACAGCAGATTCGCCTGGATGACCAGAAAGAGCGAGGAAACCATCACCCCCTGGAACTTCGAGGAAGTGGTGGAC
AAGGGCGCTTCCGCCCAGAGCTTCATCGAGCGGATGACCAACTTCGATAAGAACCTGCCCAACGAGAAGGTGCTGCCCAAGCACAGCCTG
CTGTACGAGTACTTCACCGTGTATAACGAGCTGACCAAAGTGAAATACGTGACCGAGGGAATGAGAAAGCCCGCCTTCCTGAGCGGCGAG
CAGAAAAAGGCCATCGTGGACCTGCTGTTCAAGACCAACCGGAAAGTGACCGTGAAGCAGCTGAAAGAGGACTACTTCAAGAAAATCGAG
TGCTTCGACTCCGTGGAAATCTCCGGCGTGGAAGATCGGTTCAACGCCTCCCTGGGCACATACCACGATCTGCTGAAAATTATCAAGGAC
AAGGACTTCCTGGACAATGAGGAAAACGAGGACATTCTGGAAGATATCGTGCTGACCCTGACACTGTTTGAGGACAGAGAGATGATCGAG
GAACGGCTGAAAACCTATGCCCACCTGTTCGACGACAAAGTGATGAAGCAGCTGAAGCGGCGGAGATACACCGGCTGGGGCAGGCTGAGC
CGGAAGCTGATCAACGGCATCCGGGACAAGCAGTCCGGCAAGACAATCCTGGATTTCCTGAAGTCCGACGGCTTCGCCAACAGAAACTTC
ATGCAGCTGATCCACGACGACAGCCTGACCTTTAAAGAGGACATCCAGAAAGCCCAGGTGTCCGGCCAGGGCGATAGCCTGCACGAGCAC
ATTGCCAATCTGGCCGGCAGCCCCGCCATTAAGAAGGGCATCCTGCAGACAGTGAAGGTGGTGGACGAGCTCGTGAAAGTGATGGGCCGG
CACAAGCCCGAGAACATCGTGATCGAAATGGCCAGAGAGAACCAGACCACCCAGAAGGGACAGAAGAACAGCCGCGAGAGAATGAAGCGG
ATCGAAGAGGGCATCAAAGAGCTGGGCAGCCAGATCCTGAAAGAACACCCCGTGGAAAACACCCAGCTGCAGAACGAGAAGCTGTACCTG
TACTACCTGCAGAATGGGCGGGATATGTACGTGGACCAGGAACTGGACATCAACCGGCTGTCCGACTACGATGTGGACGCTATCGTGCCT
CAGAGCTTTCTGAAGGACGACTCCATCGACAACAAGGTGCTGACCAGAAGCGACAAGAACCGGGGCAAGAGCGACAACGTGCCCTCCGAA
GAGGTCGTGAAGAAGATGAAGAACTACTGGCGGCAGCTGCTGAACGCCAAGCTGATTACCCAGAGAAAGTTCGACAATCTGACCAAGGCC
GAGAGAGGCGGCCTGAGCGAACTGGATAAGGCCGGCTTCATCAAGAGACAGCTGGTGGAAACCCGGCAGATCACAAAGCACGTGGCACAG
ATCCTGGACTCCCGGATGAACACTAAGTACGACGAGAATGACAAGCTGATCCGGGAAGTGAAAGTGATCACCCTGAAGTCCAAGCTGGTG
TCCGATTTCCGGAAGGATTTCCAGTTTTACAAAGTGCGCGAGATCAACAACTACCACCACGCCCACGACGCCTACCTGAACGCCGTCGTG
GGAACCGCCCTGATCAAAAAGTACCCTAAGCTGGAAAGCGAGTTCGTGTACGGCGACTACAAGGTGTACGACGTGCGGAAGATGATCGCC
AAGAGCGAGCAGGAAATCGGCAAGGCTACCGCCAAGTACTTCTTCTACAGCAACATCATGAACTTTTTCAAGACCGAGATTACCCTGGCC
AACGGCGAGATCCGGAAGCGGCCTCTGATCGAGACAAACGGCGAAACCGGGGAGATCGTGTGGGATAAGGGCCGGGATTTTGCCACCGTG
CGGAAAGTGCTGAGCATGCCCCAAGTGAATATCGTGAAAAAGACCGAGGTGCAGACAGGCGGCTTCAGCAAAGAGTCTATCCTGCCCAAG
AGGAACAGCGATAAGCTGATCGCCAGAAAGAAGGACTGGGACCCTAAGAAGTACGGCGGCTTCGACAGCCCCACCGTGGCCTATTCTGTG

CTGGTGGTGGCCAAAGTGGAAAAGGGCAAGTCCAAGAAACTGAAGAGTGTGAAAGAGCTGCTGGGGATCACCATCATGGAAAGAAGCAGC
TTCGAGAAGAATCCCATCGACTTTCTGGAAGCCAAGGGCTACAAAGAAGTGAAAAAGGACCTGATCATCAAGCTGCCTAAGTACTCCCTG
TTCGAGCTGGAAAACGGCCGGAAGAGAATGCTGGCCTCTGCCGGCGAACTGCAGAAGGGAAACGAACTGGCCCTGCCCTCCAAATATGTG
AACTTCCTGTACCTGGCCAGCCACTATGAGAAGCTGAAGGGCTCCCCCGAGGATAATGAGCAGAAACAGCTGTTTGTGGAACAGCACAAG
CACTACCTGGACGAGATCATCGAGCAGATCAGCGAGTTCTCCAAGAGAGTGATCCTGGCCGACGCTAATCTGGACAAAGTGCTGTCCGCC
TACAACAAGCACCGGGATAAGCCCATCAGAGAGCAGGCCGAGAATATCATCCACCTGTTTACCCTGACCAATCTGGGAGCCCCTGCCGCC
TTCAAGTACTTTGACACCACCATCGACCGGAAGAGGTACACCAGCACCAAAGAGGTGCTGGACGCCACCCTGATCCACCAGAGCATCACC
GGCCTGTACGAGACACGGATCGACCTGTCTCAGCTGGGAGGTGACTCTGGCGGCTCAAAAAGAACCGCCGACGGCAGCGAATTCGAGTCT
CCCAAGAAGAAGAGGAAAGTCGGCTCTGGCCCTGCCGCTAAGAGAGTGAAGCTGGACTAA

Sequence of MCP–RT(PE6c) used for petRNA experiments

MCP-RT(PE6c) created from previously described MCP-RT[2] and PE6c[3] constructs. M-MLV reverse transcriptase from MCP-RT (Addgene #181799) was replaced with the reverse transcriptase from PE6c.

CMV enhancer-CMV promoter-T7 promoter-MS2-N55K-linker-PE6c reverse transcriptase-SV40 NLS

GACATTGATTATTGACTAGTTATTAATAGTAATCAATTACGGGGTCATTAGTTCATAGCCCATATATGGAGTTCCGCGTTACATAA
CTTACGGTAAATGGCCCGCCTGGCTGACCGCCCAACGACCCCCGCCCATTGACGTCAATAATGACGTATGTTCCCATAGTAACGCC
AATAGGGACTTTCCATTGACGTCAATGGGTGGAGTATTTACGGTAAACTGCCCACTTGGCAGTACATCAAGTGTATCATATGCCAA
GTACGCCCCCTATTGACGTCAATGACGGTAAATGGCCCGCCTGGCATTATGCCCAGTACATGACCTTATGGGACTTTCCTACTTGG
CAGTACATCTACGTATTAGTCATCGCTATTACCATGGTGATGCGGTTTTGGCAGTACATCAATGGGCGTGGATAGCGGTTTGACTC
ACGGGGATTTCCAAGTCTCCACCCCATTGACGTCAATGGGAGTTTGTTTTGGCACCAAAATCAACGGGACTTTCCAAAATGTCGTA
ACAACTCCGCCCCATTGACGCAAATGGGCGGTAGGCGTGTACGGTGGGAGGTCTATATAAGCAGAGCTGGTTTAGTGAACCGTCAG
ATCCGCTAGAGATCCGCGGCCGCTAATACGACTCACTATAGGGAGAGCCGCCACCATGGCTTCAAACTTTACTCAGTTCGTGCTCG
TGGACAATGGTGGGACAGGGGATGTGACAGTGGCTCCTTCTAATTTCGCTAATGGGGTGGCAGAGTGGATCAGCTCCAACTCACGG
AGCCAGGCCTACAAGGTGACATGCAGCGTCAGGCAGTCTAGTGCCCAGAAGAGAAAGTATACCATCAAGGTGGAGGTCCCCAAAGT
GGCTACCCAGACAGTGGGCGGAGTCGAACTGCCTGTCGCCGCTTGGAGGTCCTACCTGAACATGGAGCTCACTATCCCAATTTTCG
CTACCAATTCTGACTGTGAACTCATCGTGAAGGCAATGCAGGGGCTCCTCAAAGACGGTAATCCTATCCCTTCCGCCATCGCCGCT
AACTCAGGTATCTACTCTGGAGGATCTAGCGGAGGATCCTCTGGCAGCGAGACACCAGGAACAAGCGAGTCAGCAACACCAGAGAG
CAGTGGCGGCAGCAGCGGCGGCAGCAGCATCAGCAGCTCTAAGCACACCCTGAGCCAGATGAACAAGGTGAGCAACATCGTGAAGG
AACCCGAGCTGCCCGACATCTACAAGGAATTTAAGGACATCACCGCCGACACCAATACCGAGAAGCTGCCTAAGCCTATTAAGGGC
CTGGAATTTGAAGTGGAACTGACACAGGAGAACTACAGACTGCCTATCCGGAACTATCCTCTGACTCCAGTCAAGATGCAGGCCAT
GAACGACGAGATCAATCAAGGCCTGAAAGGCGGCATCATCAGAGAGAGCAAGGCCATCAACGCCTGCCCTGTTATATTCGTGCCCA
GAAAGGAAGGCACACTGCGCATGGTGGTCGACTACAGGCCCCTGAACAAGTACGTGAAGCCCAACGTCTACCCCCTGCCACTGATT
GAACAACTGCTGGCCAAGATCCAGGGCAGCACCATTTTCACCAAGCTGGACCTGAAAAGCGCCTACCACCAGATCAGAGTGCGAAA
GGGAGATGAGCACAAGCTGGCCTTCCGGTGCCCCAGAGGAGTCTTCGAGTACCTGGTGATGCCTTACGGCATCAAGACAGCCCCTG
CCCACTTTCAGTACTTCATCAACACAATCCTGGGCGAGGCCAAGGAAAGCCACGTGGTGTGCTACATGGACGACATCCTGATCCAT
TCCAAGTCCGAGTCCGAACACGTGAAACATGTGAAGGACGTGCTGCAAAAGCTGAAGAACGCTAATCTGATCATCAACCAGGCCAA
ATGCGAGTTTCACCAGAGCCAAGTGAAGTTCCTGGGCTACCACATCAGCGAGAAGGGCTTAACCCCATGTCAGGAGAACATCGACA
AGGTGCTGCAGTGGAAACAGCCTAAAAACCAGAAGGAACTGAGACAGTTCCTGGGCCAGGTGAACTACCTGAGAAAATTCATCCCC
AAAACCAGCCAGTTGACCCACCCTCTGAACAAACTCCTGAAAAAGGATGTCAGATGGAAATGGACCCCTACCCAGACCCAGGCTAT
CGAGAATATCAAGCAATGTCTGGTGTCTCCTCCTGTGCTGAGGCACTTCGACTTCAGCAAGAAGATCCTGCTTGAGACAGACGTTT
CTGATGTGGCCGTGGGAGCCGTGCTGAGCCAGAAGCATGATGATGATAAGTACTACCCTGTGGGCTATTACAGCGCTAAAATGAGC
AAAGCCCAGCTGAATTATTCTGTGTCCGACAAGGAGATGCTGGCTATCATCAAAAGCCTGGAGCACTGGCGGCACTACCTGGAATC
TACAATCGAGCCCTTCAAGATCCTGACCGACCACAGAAACCTGATTGGAAGAATCACAAACGAGAGCGAACCAGAGAACAAGCGGC
TGGCCAGATGGCAGCTGTTCCTGCAGGACTTCAACTTCGAGATCAACTACAGACCTGGCTCTGCAAATCACATCGCCGATGCCCTG
TCTAGAATCGTGGACGAGACTGAGCCTATCCCTAAGGACAACGAAGATAACAGCATCAACTTCGTGAACCAGATCAGCATCCCCAA
GAAGAAGAGGAAAGTCTAA

Sequence of PE6c-P2A-EGFP plasmid used for HEK293T cell FACS experiments from Extended Data Figure 14.

The T7 promoter within this construct has a mismatch to facilitate generation of linear DNA template for in vitro transcription, as previously described[4]. This plasmid is suitable for standard lipid-based transfection or as PCR template to generate IVT template.

CMV enhancer-CMV promoter-mismatched T7 promoter-BPNLS-SV40 NLS-Cas9(R221K N394K H840A)- BPNLS-SV40 NLS-PE6c reverse transcriptase- BPNLS-SV40 NLS-GSG-cmycNLS-P2A element-EGFP-SV40 NLS

GACATTGATTATTGACTAGTTATTAATAGTAATCAATTACGGGGTCATTAGTTCATAGCCCATATATGGAGTTCCGCGTTACATAACTTA
CGGTAAATGGCCCGCCTGGCTGACCGCCCAACGACCCCCGCCCATTGACGTCAATAATGACGTATGTTCCCATAGTAACGCCAATAGGGA
CTTTCCATTGACGTCAATGGGTGGAGTATTTACGGTAAACTGCCCACTTGGCAGTACATCAAGTGTATCATATGCCAAGTACGCCCCCTA
TTGACGTCAATGACGGTAAATGGCCCGCCTGGCATTATGCCCAGTACATGACCTTATGGGACTTTCCTACTTGGCAGTACATCTACGTAT
TAGTCATCGCTATTACCATGGTGATGCGGTTTTGGCAGTACATCAATGGGCGTGGATAGCGGTTTGACTCACGGGGATTTCCAAGTCTCC
ACCCCATTGACGTCAATGGGAGTTTGTTTTGGCACCAAAATCAACGGGACTTTCCAAAATGTCGTAACAACTCCGCCCCATTGACGCAAA
TGGGCGGTAGGCGTGTACGGTGGGAGGTCTATATAAGCAGAGCTGGTTTAGTGAACCGTCAGATCTCGAGCTCGGTACCTAATACGACAC
ACTATAAGGAAATAAGAGAGAAAAGAAGAGTAAGAAGAAATATAAGAGCCACCATGAAACGGACAGCCGACGGAAGCGAGTTCGAGTCAC
CAAAGAAGAAGCGGAAAGTCGACAAGAAGTACAGCATCGGCCTGGACATCGGCACCAACTCTGTGGGCTGGGCCGTGATCACCGACGAGT
ACAAGGTGCCCAGCAAGAAATTCAAGGTGCTGGGCAACACCGACCGGCACAGCATCAAGAAGAACCTGATCGGAGCCCTGCTGTTCGACA
GCGGCGAAACAGCCGAGGCCACCCGGCTGAAGAGAACCGCCAGAAGAAGATACACCAGACGGAAGAACCGGATCTGCTATCTGCAAGAGA
TCTTCAGCAACGAGATGGCCAAGGTGGACGACAGCTTCTTCCACAGACTGGAAGAGTCCTTCCTGGTGGAAGAGGATAAGAAGCACGAGC
GGCACCCCATCTTCGGCAACATCGTGGACGAGGTGGCCTACCACGAGAAGTACCCCACCATCTACCACCTGAGAAAGAAACTGGTGGACA
GCACCGACAAGGCCGACCTGCGGCTGATCTATCTGGCCCTGGCCCACATGATCAAGTTCCGGGGCCACTTCCTGATCGAGGGCGACCTGA
ACCCCGACAACAGCGACGTGGACAAGCTGTTCATCCAGCTGGTGCAGACCTACAACCAGCTGTTCGAGGAAAACCCCATCAACGCCAGCG
GCGTGGACGCCAAGGCCATCCTGTCTGCCAGACTGAGCAAGAGCAGAAAGCTGGAAAATCTGATCGCCCAGCTGCCCGGCGAGAAGAAGA
ATGGCCTGTTCGGAAACCTGATTGCCCTGAGCCTGGGCCTGACCCCCAACTTCAAGAGCAACTTCGACCTGGCCGAGGATGCCAAACTGC
AGCTGAGCAAGGACACCTACGACGACGACCTGGACAACCTGCTGGCCCAGATCGGCGACCAGTACGCCGACCTGTTTCTGGCCGCCAAGA
ACCTGTCCGACGCCATCCTGCTGAGCGACATCCTGAGAGTGAACACCGAGATCACCAAGGCCCCCCTGAGCGCCTCTATGATCAAGAGAT
ACGACGAGCACCACCAGGACCTGACCCTGCTGAAAGCTCTCGTGCGGCAGCAGCTGCCTGAGAAGTACAAAGAGATTTTCTTCGACCAGA
GCAAGAACGGCTACGCCGGCTACATTGACGGCGGAGCCAGCCAGGAAGAGTTCTACAAGTTCATCAAGCCCATCCTGGAAAAGATGGACG
GCACCGAGGAACTGCTCGTGAAGCTGAAGAGAGAGGACCTGCTGCGGAAGCAGCGGACCTTCGACAACGGCAGCATCCCCCACCAGATCC
ACCTGGGAGAGCTGCACGCCATTCTGCGGCGGCAGGAAGATTTTTACCCATTCCTGAAGGACAACCGGGAAAAGATCGAGAAGATCCTGA
CCTTCCGCATCCCCTACTACGTGGGCCCTCTGGCCAGGGGAAACAGCAGATTCGCCTGGATGACCAGAAAGAGCGAGGAAACCATCACCC
CCTGGAACTTCGAGGAAGTGGTGGACAAGGGCGCTTCCGCCCAGAGCTTCATCGAGCGGATGACCAACTTCGATAAGAACCTGCCCAACG
AGAAGGTGCTGCCCAAGCACAGCCTGCTGTACGAGTACTTCACCGTGTATAACGAGCTGACCAAAGTGAAATACGTGACCGAGGGAATGA
GAAAGCCCGCCTTCCTGAGCGGCGAGCAGAAAAAGGCCATCGTGGACCTGCTGTTCAAGACCAACCGGAAAGTGACCGTGAAGCAGCTGA
AAGAGGACTACTTCAAGAAAATCGAGTGCTTCGACTCCGTGGAAATCTCCGGCGTGGAAGATCGGTTCAACGCCTCCCTGGGCACATACC
ACGATCTGCTGAAAATTATCAAGGACAAGGACTTCCTGGACAATGAGGAAAACGAGGACATTCTGGAAGATATCGTGCTGACCCTGACAC
TGTTTGAGGACAGAGAGATGATCGAGGAACGGCTGAAAACCTATGCCCACCTGTTCGACGACAAAGTGATGAAGCAGCTGAAGCGGCGGA
GATACACCGGCTGGGGCAGGCTGAGCCGGAAGCTGATCAACGGCATCCGGGACAAGCAGTCCGGCAAGACAATCCTGGATTTCCTGAAGT
CCGACGGCTTCGCCAACAGAAACTTCATGCAGCTGATCCACGACGACAGCCTGACCTTTAAAGAGGACATCCAGAAAGCCCAGGTGTCCG
GCCAGGGCGATAGCCTGCACGAGCACATTGCCAATCTGGCCGGCAGCCCCGCCATTAAGAAGGGCATCCTGCAGACAGTGAAGGTGGTGG
ACGAGCTCGTGAAAGTGATGGGCCGGCACAAGCCCGAGAACATCGTGATCGAAATGGCCAGAGAGAACCAGACCACCCAGAAGGGACAGA
AGAACAGCCGCGAGAGAATGAAGCGGATCGAAGAGGGCATCAAAGAGCTGGGCAGCCAGATCCTGAAAGAACACCCCGTGGAAAACACCC
AGCTGCAGAACGAGAAGCTGTACCTGTACTACCTGCAGAATGGGCGGGATATGTACGTGGACCAGGAACTGGACATCAACCGGCTGTCCG
ACTACGATGTGGACGCTATCGTGCCTCAGAGCTTTCTGAAGGACGACTCCATCGACAACAAGGTGCTGACCAGAAGCGACAAGAACCGGG
GCAAGAGCGACAACGTGCCCTCCGAAGAGGTCGTGAAGAAGATGAAGAACTACTGGCGGCAGCTGCTGAACGCCAAGCTGATTACCCAGA
GAAAGTTCGACAATCTGACCAAGGCCGAGAGAGGCGGCCTGAGCGAACTGGATAAGGCCGGCTTCATCAAGAGACAGCTGGTGGAAACCC
GGCAGATCACAAAGCACGTGGCACAGATCCTGGACTCCCGGATGAACACTAAGTACGACGAGAATGACAAGCTGATCCGGGAAGTGAAAG
TGATCACCCTGAAGTCCAAGCTGGTGTCCGATTTCCGGAAGGATTTCCAGTTTTACAAAGTGCGCGAGATCAACAACTACCACCACGCCC
ACGACGCCTACCTGAACGCCGTCGTGGGAACCGCCCTGATCAAAAAGTACCCTAAGCTGGAAAGCGAGTTCGTGTACGGCGACTACAAGG
TGTACGACGTGCGGAAGATGATCGCCAAGAGCGAGCAGGAAATCGGCAAGGCTACCGCCAAGTACTTCTTCTACAGCAACATCATGAACT
TTTTCAAGACCGAGATTACCCTGGCCAACGGCGAGATCCGGAAGCGGCCTCTGATCGAGACAAACGGCGAAACCGGGGAGATCGTGTGGG

ATAAGGGCCGGGATTTTGCCACCGTGCGGAAAGTGCTGAGCATGCCCCAAGTGAATATCGTGAAAAAGACCGAGGTGCAGACAGGCGGCT
TCAGCAAAGAGTCTATCCTGCCCAAGAGGAACAGCGATAAGCTGATCGCCAGAAAGAAGGACTGGGACCCTAAGAAGTACGGCGGCTTCG
ACAGCCCCACCGTGGCCTATTCTGTGCTGGTGGTGGCCAAAGTGGAAAAGGGCAAGTCCAAGAAACTGAAGAGTGTGAAAGAGCTGCTGG
GGATCACCATCATGGAAAGAAGCAGCTTCGAGAAGAATCCCATCGACTTTCTGGAAGCCAAGGGCTACAAAGAAGTGAAAAAGGACCTGA
TCATCAAGCTGCCTAAGTACTCCCTGTTCGAGCTGGAAAACGGCCGGAAGAGAATGCTGGCCTCTGCCGGCGAACTGCAGAAGGGAAACG
AACTGGCCCTGCCCTCCAAATATGTGAACTTCCTGTACCTGGCCAGCCACTATGAGAAGCTGAAGGGCTCCCCCGAGGATAATGAGCAGA
AACAGCTGTTTGTGGAACAGCACAAGCACTACCTGGACGAGATCATCGAGCAGATCAGCGAGTTCTCCAAGAGAGTGATCCTGGCCGACG
CTAATCTGGACAAAGTGCTGTCCGCCTACAACAAGCACCGGGATAAGCCCATCAGAGAGCAGGCCGAGAATATCATCCACCTGTTTACCC
TGACCAATCTGGGAGCCCCTGCCGCCTTCAAGTACTTTGACACCACCATCGACCGGAAGAGGTACACCAGCACCAAAGAGGTGCTGGACG
CCACCCTGATCCACCAGAGCATCACCGGCCTGTACGAGACACGGATCGACCTGTCTCAGCTGGGAGGTGACTCCGGCGGAAGCTCTGGTG
GCAGCAAGCGGACCGCCGACGGCTCTGAATTCGAGAGCCCTAAGAAGAAAAGAAAGGTGAGCGGAGGCTCTAGCGGCGGAAGCATCAGCA
GCTCTAAGCACACCCTGAGCCAGATGAACAAGGTGAGCAACATCGTGAAGGAACCCGAGCTGCCCGACATCTACAAGGAATTTAAGGACA
TCACCGCCGACACCAATACCGAGAAGCTGCCTAAGCCTATTAAGGGCCTGGAATTTGAAGTGGAACTGACACAGGAGAACTACAGACTGC
CTATCCGGAACTATCCTCTGACTCCAGTCAAGATGCAGGCCATGAACGACGAGATCAATCAAGGCCTGAAAGGCGGCATCATCAGAGAGA
GCAAGGCCATCAACGCCTGCCCTGTTATATTCGTGCCCAGAAAGGAAGGCACACTGCGCATGGTGGTCGACTACAGGCCCCTGAACAAGT
ACGTGAAGCCCAACGTCTACCCCCTGCCACTGATTGAACAACTGCTGGCCAAGATCCAGGGCAGCACCATTTTCACCAAGCTGGACCTGA
AAAGCGCCTACCACCAGATCAGAGTGCGAAAGGGAGATGAGCACAAGCTGGCCTTCCGGTGCCCCAGAGGAGTCTTCGAGTACCTGGTGA
TGCCTTACGGCATCAAGACAGCCCCTGCCCACTTTCAGTACTTCATCAACACAATCCTGGGCGAGGCCAAGGAAAGCCACGTGGTGTGCT
ACATGGACGACATCCTGATCCATTCCAAGTCCGAGTCCGAACACGTGAAACATGTGAAGGACGTGCTGCAAAAGCTGAAGAACGCTAATC
TGATCATCAACCAGGCCAAATGCGAGTTTCACCAGAGCCAAGTGAAGTTCCTGGGCTACCACATCAGCGAGAAGGGCTTAACCCCATGTC
AGGAGAACATCGACAAGGTGCTGCAGTGGAAACAGCCTAAAAACCAGAAGGAACTGAGACAGTTCCTGGGCCAGGTGAACTACCTGAGAA
AATTCATCCCCAAAACCAGCCAGTTGACCCACCCTCTGAACAAACTCCTGAAAAAGGATGTCAGATGGAAATGGACCCCTACCCAGACCC
AGGCTATCGAGAATATCAAGCAATGTCTGGTGTCTCCTCCTGTGCTGAGGCACTTCGACTTCAGCAAGAAGATCCTGCTTGAGACAGACG
TTTCTGATGTGGCCGTGGGAGCCGTGCTGAGCCAGAAGCATGATGATGATAAGTACTACCCTGTGGGCTATTACAGCGCTAAAATGAGCA
AAGCCCAGCTGAATTATTCTGTGTCCGACAAGGAGATGCTGGCTATCATCAAAAGCCTGGAGCACTGGCGGCACTACCTGGAATCTACAA
TCGAGCCCTTCAAGATCCTGACCGACCACAGAAACCTGATTGGAAGAATCACAAACGAGAGCGAACCAGAGAACAAGCGGCTGGCCAGAT
GGCAGCTGTTCCTGCAGGACTTCAACTTCGAGATCAACTACAGACCTGGCTCTGCAAATCACATCGCCGATGCCCTGTCTAGAATCGTGG
ACGAGACTGAGCCTATCCCTAAGGACAACGAAGATAACAGCATCAACTTCGTGAACCAGATCAGCATCTCTGGCGGCTCAAAAAGAACCG
CCGACGGCAGCGAATTCGAGTCTCCCAAGAAGAAGAGGAAAGTCGGCTCTGGCCCTGCCGCTAAGAGAGTGAAGCTGGACGGAAGCGGAG
CTACTAACTTCAGCCTGCTGAAGCAGGCTGGAGACGTGGAGGAGAACCCTGGACCTATGGTGAGCAAGGGCGAGGAGCTGTTCACCGGGG
TGGTGCCCATCCTGGTCGAGCTGGACGGCGACGTAAACGGCCACAAGTTCAGCGTGTCCGGCGAGGGCGAGGGCGATGCCACCTACGGCA
AGCTGACCCTGAAGTTCATCTGCACCACCGGCAAGCTGCCCGTGCCCTGGCCCACCCTCGTGACCACCCTGACCTACGGCGTGCAGTGCT
TCAGCCGCTACCCCGACCACATGAAGCAGCACGACTTCTTCAAGTCCGCCATGCCCGAAGGCTACGTCCAGGAGCGCACCATCTTCTTCA
AGGACGACGGCAACTACAAGACCCGCGCCGAGGTGAAGTTCGAGGGCGACACCCTGGTGAACCGCATCGAGCTGAAGGGCATCGACTTCA
AGGAGGACGGCAACATCCTGGGGCACAAGCTGGAGTACAACTACAACAGCCACAACGTCTATATCATGGCCGACAAGCAGAAGAACGGCA
TCAAGGTGAACTTCAAGATCCGCCACAACATCGAGGACGGCAGCGTGCAGCTCGCCGACCACTACCAGCAGAACACCCCCATCGGCGACG
GCCCCGTGCTGCTGCCCGACAACCACTACCTGAGCACCCAGTCCGCCCTGAGCAAAGACCCCAACGAGAAGCGCGATCACATGGTCCTGC
TGGAGTTCGTGACCGCCGCCGGGATCACTCTCGGCATGGACGAGCTGTACAAGTCTGGTGGTTCTCCTAAGAAAAAGCGGAAGGTGTAA

Supplementary Note 1 | Python script used for quantification of scaffold incorporation.

```
# pegHunt
# Adapted from Anzalone et al., Nature, 2019
# Note to user: Save and run script as Jupyter Notebook (.ipynb) file
#!/usr/bin/env python
# coding: utf-8

# In[1]:



import pandas as pd
import Bio as bio
from Bio import SeqIO
import glob



# In[2]:



sources = glob.glob('*.fastq')



# In[3]:



fastqdict = {}
for i in range(len(sources)):
    temp = list(SeqIO.parse(sources[i],"fastq"))
    fastqdict[sources[i]]= [str(temp[k].seq) for k in range(len(temp))]
print(len(sources))



# In[4]:



scaffdict =
{'P':'TCTGTATCTATATTCATCATGTTTTAGAGCTAGAAATAGCAAGTTAAAATAAGGCTAGTCCGTTATCAACTTGAAAAAGTGGCA
CCGAGTCGGTGCTGGCACCATTAAAGAAAATATCATCTTTGGTGTGAGTTACG'}
```

```python
RTTlen = 41
L = len(scaffdict['P'])
print(L)
print(scaffdict['P'][(L-RTTlen):])
print(scaffdict['P'][(L-RTTlen-1):])


# In[5]:


resultdict = dict.fromkeys(sources)
for key in fastqdict:
    for scaffold in scaffdict:
        if scaffold in str(key):
            resultlist = []
            for j in range(L-RTTlen):
                #extent = scaffdict[scaffold][0:(j+1)]
                extent = scaffdict[scaffold][(L-RTTlen-j):]
                if j < 5:
                    print(extent)
                counter = 0
                for i in range(len(fastqdict[key])):
                    if extent in fastqdict[key][i]:
                        counter = counter + 1
                resultlist.append(counter)
            resultdict[key]=resultlist


# In[6]:


resultdf = pd.DataFrame({k: pd.Series(v) for k, v in resultdict.items()})
resultdf = resultdf.reindex(sorted(resultdf.columns), axis=1)
resultdf = resultdf.fillna(0.0)
resultdf.index = range(1,len(resultdf)+1)


# In[7]:
```

```
resultdf2=resultdf.copy()
for i in range(1,len(resultdf)):
    resultdf2.loc[i] = resultdf.loc[i].subtract(resultdf.loc[i+1])


# In[8]:


resultdf3=resultdf2.copy()
for entry in resultdf3:
    resultdf3[entry] = resultdf2[entry].div(resultdf[entry][1])*100


# In[9]:


resultdf.to_excel('cumulativeincorp_RC1_wLAS288.xlsx')


# In[10]:


resultdf2.to_excel('specificbasesincorporated2_RC1_wLAS288.xlsx')


# In[11]:


resultdf3.to_excel('incorpfreq_RC1_wLAS288.xlsx')
```

Supplementary References

1. Chen, P. J. *et al.* Enhanced prime editing systems by manipulating cellular determinants of editing outcomes. *Cell* **184**, 5635-5652.e29 (2021).
2. Liu, B. *et al.* A split prime editor with untethered reverse transcriptase and circular RNA template. *Nat. Biotechnol.* **40**, 1388–1393 (2022).
3. Doman, J. L. *et al.* Phage-assisted evolution and protein engineering yield compact, efficient prime editors. *Cell* **186**, 3983-4002.e26 (2023).
4. Doman, J. L., Sousa, A. A., Randolph, P. B., Chen, P. J. & Liu, D. R. Designing and executing prime editing experiments in mammalian cells. *Nat. Protoc.* 1–38 (2022) doi:10.1038/s41596-022-00724-4.
