# Supplementary material for: Systematic optimization of prime editing for the efficient functional correction of CFTR F508del in human airway epithelial cells
Source: Nat Biomed Eng. 2024 Jul 10;9(1):7–21. doi: 10.1038/s41551-024-01233-3 (PMC11754097; doi:10.1038/s41551-024-01233-3)
Supplement: Supplementary file 2 — Reporting Summary [file 41551_2024_1233_MOESM2_ESM.pdf]

nature portfolio

Corresponding author(s): David R. Liu

Last updated by author(s): May 21, 2024

Reporting Summary

Nature Portfolio wishes to improve the reproducibility of the work that we publish. This form provides structure for consistency and transparency in reporting. For further information on Nature Portfolio policies, see our Editorial Policies and the Editorial Policy Checklist.

Statistics

For all statistical analyses, confirm that the following items are present in the figure legend, table legend, main text, or Methods section.

n/a	Confirmed	
☐	☒	The exact sample size (*n*) for each experimental group/condition, given as a discrete number and unit of measurement
☐	☒	A statement on whether measurements were taken from distinct samples or whether the same sample was measured repeatedly
☐	☒	The statistical test(s) used AND whether they are one- or two-sided<br>*Only common tests should be described solely by name; describe more complex techniques in the Methods section.*
☐	☒	A description of all covariates tested
☐	☒	A description of any assumptions or corrections, such as tests of normality and adjustment for multiple comparisons
☐	☒	A full description of the statistical parameters including central tendency (e.g. means) or other basic estimates (e.g. regression coefficient) AND variation (e.g. standard deviation) or associated estimates of uncertainty (e.g. confidence intervals)
☐	☒	For null hypothesis testing, the test statistic (e.g. *F*, *t*, *r*) with confidence intervals, effect sizes, degrees of freedom and *P* value noted<br>*Give P values as exact values whenever suitable.*
☒	☐	For Bayesian analysis, information on the choice of priors and Markov chain Monte Carlo settings
☒	☐	For hierarchical and complex designs, identification of the appropriate level for tests and full reporting of outcomes
☒	☐	Estimates of effect sizes (e.g. Cohen's *d*, Pearson's *r*), indicating how they were calculated

Our web collection on statistics for biologists contains articles on many of the points above.

Software and code

Policy information about availability of computer code

Data collection	Illumina Miseq Control software (3.1) was used on the Illumina Miseq sequencers to collect the high-throughput sequencing data. Sony MA900 sorter was used for FACS with MA900 Cell Sorter software v3.1.
Data analysis	CRISPResso2 was used to analyse Miseq data for quantifying %edit and %indels at genomic loci; CRISPRess2 is documented on GitHub. Custom Python code to assess pegRNA scaffold insertion is provided as Supplementary Information. FlowJo 10.10.0 was used to analyse FACS data.

For manuscripts utilizing custom algorithms or software that are central to the research but not yet described in published literature, software must be made available to editors and reviewers. We strongly encourage code deposition in a community repository (e.g. GitHub). See the Nature Portfolio guidelines for submitting code & software for further information.

Data

Policy information about availability of data

All manuscripts must include a data availability statement. This statement should provide the following information, where applicable:

- Accession codes, unique identifiers, or web links for publicly available datasets
- A description of any restrictions on data availability
- For clinical datasets or third party data, please ensure that the statement adheres to our policy

All data supporting the results of this study are available within the paper and its Supplementary Information. High-throughput sequencing data are available from

the NCBI Sequence Read Archive database (PRJNA1055086). Source data for the figures are provided. Key plasmids are available from Addgene (depositor: David R. Liu), or from the corresponding author on request.

Research involving human participants, their data, or biological material

Policy information about studies with human participants or human data. See also policy information about sex, gender (identity/presentation), and sexual orientation and race, ethnicity and racism.

Reporting on sex and gender	The researchers were blinded to the sex and gender identities of the biological material.
Reporting on race, ethnicity, or other socially relevant groupings	The researchers were blinded to the race, ethnicity, and other socially relevant groupings of the biological material.
Population characteristics	The researchers were blinded to the population characteristics of the biological material.
Recruitment	Primary airway epithelial cells from non-CF donors were isolated from trachea or bronchi, from post-mortem lungs that were unsuitable for transplantation. Primary CF airway epithelial cells were isolated from lung tissue obtained following lung transplant.
Ethics oversight	The studies were approved by the University of Iowa Institutional Review Board, under United States Department of Health and Human Services registration number IRB00000099.

Note that full information on the approval of the study protocol must also be provided in the manuscript.

Field-specific reporting

Please select the one below that is the best fit for your research. If you are not sure, read the appropriate sections before making your selection.

[X] Life sciences [ ] Behavioural & social sciences [ ] Ecological, evolutionary & environmental sciences

For a reference copy of the document with all sections, see nature.com/documents/nr-reporting-summary-flat.pdf

Life sciences study design

All studies must disclose on these points even when the disclosure is negative.

Sample size	Sample sizes were determined on the basis of literature precedents for genome-editing experiments (for example, Anzalone et al., Nature 2019).
Data exclusions	No data were excluded.
Replication	All experiments described in the main text and in the Extended data figures were performed with three replicates, and all attempts at replication were successful.
Randomization	The mammalian cells used in this study were grown under identical conditions; no randomization was used.
Blinding	The mammalian cells used in this study were grown under identical conditions; blinding was not used.

Reporting for specific materials, systems and methods

We require information from authors about some types of materials, experimental systems and methods used in many studies. Here, indicate whether each material, system or method listed is relevant to your study. If you are not sure if a list item applies to your research, read the appropriate section before selecting a response.

Materials & experimental systems

n/a	Involved in the study	
X		Antibodies
	X	Eukaryotic cell lines
X		Palaeontology and archaeology
X		Animals and other organisms
X		Clinical data
X		Dual use research of concern
X		Plants

Methods

n/a	Involved in the study	
X		ChIP-seq
	X	Flow cytometry
X		MRI-based neuroimaging

Eukaryotic cell lines

Policy information about cell lines and Sex and Gender in Research

Cell line source(s)	HEK293T (ATCC, female); 16HBEge-F508del (a gift from Cystic Fibrosis Foundation, male); primary CF and non-CF airway epithelial cells (obtained from the Iowa Donor Network; the researchers were blinded to the sex identities of the primary cell lines).
Authentication	HEK293T, 16HBEge-F508del and primary CF and non-CF epithelial cells were authenticated by the supplier.
Mycoplasma contamination	All cell lines tested negative for mycoplasma.
Commonly misidentified lines (See ICLAC register)	No commonly misidentified cell lines were used.

Flow Cytometry

Plots

Confirm that:

☒ The axis labels state the marker and fluorochrome used (e.g. CD4-FITC).

☒ The axis scales are clearly visible. Include numbers along axes only for bottom left plot of group (a 'group' is an analysis of identical markers).

☒ All plots are contour plots with outliers or pseudocolor plots.

☒ A numerical value for number of cells or percentage (with statistics) is provided.

Methodology

Sample preparation	The preparation of the HEK293T cells for FACS is described in Methods.
Instrument	Sony MA900 Cell Sorter (Sony Biotechnology)
Software	MA900 Cell Sorter software v3.1
Cell population abundance	Between 0–30% of sorted cells were GFP-positive.
Gating strategy	HEK293T cells were first gated (HEK293T Cells gate) based on forward (FSC-A) and side scattering (SSC-A) to remove dead cells and other debris. A second gate (Single Cells gate) was used to select singlets based on FSC-H and FSC-A. Finally, a gate to collect all cells (Bulk Cells gate) and a gate to collect GFP positive cells (GFP+ gate) were analysed via the EGFP-A (FITC) channel, and sorted into samples of at least 100,000 cells. The gating strategy is provided in Supplementary Fig. 5.

☒ Tick this box to confirm that a figure exemplifying the gating strategy is provided in the Supplementary Information.
